# Individual and clinical variables associated with the risk of Buruli ulcer acquisition: A systematic review and meta-analysis

**DOI:** 10.1371/journal.pntd.0008161

**Published:** 2020-04-08

**Authors:** João Fevereiro, Nikta Sajjadi, Alexandra G. Fraga, Pedro M. Teixeira, Jorge Pedrosa

**Affiliations:** 1 Life and Health Sciences Research Institute (ICVS), School of Medicine, University of Minho, Braga, Portugal; 2 ICVS/3B's—PT Government Associate Laboratory, Braga/Guimarães, Portugal; Swiss Tropical and Public Health Institute, SWITZERLAND

## Abstract

**Background:**

Buruli ulcer (BU) is a necrotizing skin disease, caused by *Mycobacterium ulcerans*, with poorly understood acquisition risk factors. This review aims at evaluating the importance of individual–sex, age, family ties with history of BU, gene variants–and clinical–Bacillus Calmette-Guérin (BCG) immunization, Human Immunodeficiency Virus (HIV) infection–variables in this process.

**Methods:**

A systematic review was performed considering the following databases: ClinicalTrials.gov, Cochrane Controlled Register of Trials (CENTRAL), Current Contents Connect, Embase, MEDLINE, SciELO, Scopus and Web of Science. Eligible studies were critically appraised with The Joanna Briggs Institute checklists and heterogeneity was assessed with Cochran Q-test and *I*^*2*^ statistic. Published demographic data was descriptively analysed and clinical data pooled within random-effects modelling for meta-analysis.

**Results:**

A total of 29 studies were included in the systematic review. Two randomized controlled trials (RCTs) and 21 case-control studies were selected for meta-analysis. Studies show that BU mainly affects age extremes, more preponderately males among children. Data pooled from RCTs do not reveal BCG to be protective against BU (odds ratio (OR) = 0.63; 95% CI = 0.38–1.05; *I*^*2*^ = 56%), a finding case-control studies appear to corroborate. HIV infection (OR = 6.80; 95% CI = 2.33–19.85; *I*^*2*^ = 0%) and *SLC11A1* rs17235409 A allele (OR = 1.86; 95% CI = 1.25–2.77; *I*^*2*^ = 0%) are associated with increased prevalence of the disease. No definite conclusions can be drawn regarding the influence of previous family history of BU.

**Discussion:**

While available evidence warrants further robustness, these results have direct implications on current interventions and future research programs, and foster the development of more cost-effective preventive and screening measures.

**Registration:**

The study was registered at PROSPERO with number CRD42019123611.

## Author summary

Buruli ulcer (BU) is a devastating neglected tropical disease, whose worldwide notification to the WHO is currently on the rise. However, evidence has been so far conflicting as to the individual and clinical risk factors underlying the acquisition of the disease. Thus, considering the increasing body of literature made available in the last decade, it becomes essential to revisit this subject. To this aim, a temporal-unrestricted systematic review of published literature was undertaken in eight different online databases and meta-analysis conducted with clinical data. The randomized controlled trials and cohort studies reviewed supported the idea that male children or older adults are more susceptible to BU, BCG vaccination is insufficient to prevent BU in the mid-long term, and the prevalence of the disease is higher among HIV-infected individuals or carriers of genetic polymorphisms in *SLC11A1*. This knowledge, together with recommendations to tackle some drawbacks found within the reviewed studies, not only underlines the importance of searching for better vaccination alternatives, but also provides guidance for current and future screening and prevention programs.

## Introduction

Buruli ulcer (BU) is a neglected tropical chronic skin disease caused by *Mycobacterium ulcerans*. The archetypal BU case begins with a painless small nodule or ulcer, less commonly a plaque or edema [1]. Over a period of weeks to months, these lesions may progress to extensive ulcerative lesions, which in some circumstances can afflict the underlying bone [1]. Due to its multiple presentations and the prospect of confounding superinfections, WHO recommends, without compromising the beginning of antibiotherapy, that the diagnosis of BU should include at least one, ideally two, of the following laboratorial methods: a) direct smear examination of acid-fast bacilli (AFB) with Ziehl-Neelsen from a swab or a biopsy; b) histopathology; c) culture on Löwenstein-Jensen medium at 32ºC; and d) Polymerase Chain Reaction (PCR) targeting the IS*2404* insertion sequence [2]. The latter is currently considered the gold standard test, mostly due to its higher sensitivity and specificity (>90%), despite not being available in all laboratories [2].

BU has been reported in 34 countries worldwide [3,4]. As of 2018, there were two major foci of the disease, one mainly involving sub-Saharan African countries Ghana, Nigeria, Ivory Coast and Benin, and the other located in Australia, accounting for a total of 1892 cases, more than half of all recorded cases that year [3]. However, although there is a degree of consensus regarding the role of some environmental and behavioral factors associated to the disease, the literature has been less clear regarding the individual determinants of BU [5–7]. In fact, the only systematic reviews that have covered the intrinsic and clinical variables are either almost a decade-year old or largely inconclusive, prompting for an update on the state of the art [7,8].

Very little is known on the clinical features that predispose to BU. Sociodemographic variables, such as sex or age, have repeatedly failed to be associated to changes in the incidence of the disease, although some studies reported a higher number of cases amongst children and older adults, as well as a varying predominance of sex across the difference age groups [7,9–11]. Interestingly, while the reasons for such differences are still elusive, some studies have implicated BU as having a genetic component associated to its development. Whilst one group found that previous family history of BU significantly increased the odds for new cases in the family, other authors reported associations of alleles in *SLC11A1*, *IFNG*, amongst others, with increased chances of contracting the disease [12–15]. However, not only are many of these findings still pending validation, but others authors have also reached opposite conclusions, thus remaining the question of what role does genetics play in BU [13,16–18].

Likewise, the impact of immunological determinants, such as Bacillus Calmette-Guérin (BCG) vaccination or Human Immunodeficiency Virus (HIV) co-infection, on the development of BU is also a matter of uncertainty [19]. A recent meta-analysis concluded that although the original randomized controlled trials (RCTs) testing BCG vaccination showed a protective role for the vaccine, later case-control studies did not confirm such effect [8]. HIV co-infection, on the other hand, appears to promote the clinical manifestation of BU, but the low prevalence (<2–3%) of HIV-infected patients in many of the BU-endemic countries undermines the statistical power of the analyses and the reliability of the results [16,20,21].

Hence, considering the ambiguity governing the importance of the above-mentioned individual factors in the acquisition of BU, a systematic review of the available body of literature was herein undertaken. Eight different medical and scientific databases were screened for experimental and observational studies worldwide, from which data was extracted for meta-analysis whenever applicable. Critical analysis of such information is expected to yield valuable information on the understanding of the risk factors for BU development, with potential implications for future studies and intervention measures.

## Methods

This study complies with The Preferred Reporting Items for Systematic Reviews and Meta-Analyses statement (PRISMA; S1 Checklist) and its protocol is published and accessible at PROSPERO with the number CRD42019123611.

### Eligibility criteria

For the present systematic review and meta-analysis, data derived from human RCTs, case-control and cohort studies were considered, without any temporal or language constraints. Studies were selected if they met all of the following criteria: (1) patients with BU from any part of the world, excluding studies restricted to specific age groups or presentations of the disease to avoid selection biases, as well as RCTs with recurrent cases of BU to avoid immunological memory confounding; (2) analysis of variables age, sex, BCG vaccination, HIV status, family history of BU and gene alleles, with exclusion of methodological studies and studies mainly focusing on outcomes of the disease process, subjective perceptions or behaviors of individuals and environmental risk factors; (3) primary outcome being the acquisition of BU.

### Information sources and search

For this purpose, a literature search of the electronic databases ClinicalTrials.gov (1997 –present), Cochrane Controlled Register of Trials (CENTRAL, until present), Current Contents Connect (1998 –present, via Web of Science), Embase (1947 –present), MEDLINE (1946 –present, via PubMed), SciELO (1997 –present), Scopus (1788 –present) and Web of Science (1900 –present) was last performed on February 1, 2019, following the structure presented in the supporting information (S1 Text).

### Study selection

Eligibility assessment was performed independently by JF and NS according to the above-mentioned criteria, with disagreement between the two authors being reviewed by PT. Titles and abstracts regarded as inconclusive were considered for full text inspection. Studies were included only when all the eligibility criteria were met.

### Data collection process and summary measures

Extraction was performed by JF, NS and AGF and information independently cross-checked among these authors, namely: sample size, frequency and incidence metrics on individual characteristics of participants–age, age group, sex and genetic alleles–, as well as on the number of individuals vaccinated with BCG, infected by HIV or with previous family history of BU. Whenever the frequency of any group could not be calculated from the available data, corresponding authors were asked, via e-mail contact, to provide the necessary information.

### Statistics

Meta-analyses were conducted using Review Manager 5.3 and MetaGenyo [22,23]. Heterogeneity was tested with the Cochran Q-test and *I*^*2*^ statistic, calculated as *I*^*2*^ = [(Q–degree of freedom)/Q] × 100, where Q is the Cochran’s statistic. *I*^*2*^ values of 30, 50 and 75 represent low, medium and high heterogeneity, respectively [24]. In the presence of high heterogeneity, subgroup analysis with laboratory-BU confirmed cases and/or low risk bias studies was attempted. Random-effects model (the Restricted Maximum-Likelihood method) was used to calculate the summary of pooled frequency estimates [25].

### Risk of bias across studies

Studies were independently classified by JF and NS as to the risk of biases, using the adequate critical assessment tools developed by the Joanna Briggs Institute [26]. Whenever disagreements between authors were found, a third call to PT was solicited. In addition, meta-analyses comprising at least 10 studies were checked for the presence of potential publication bias through the visual inspection of funnel plot asymmetry.

## Results

### Study selection

**Fig 1:**
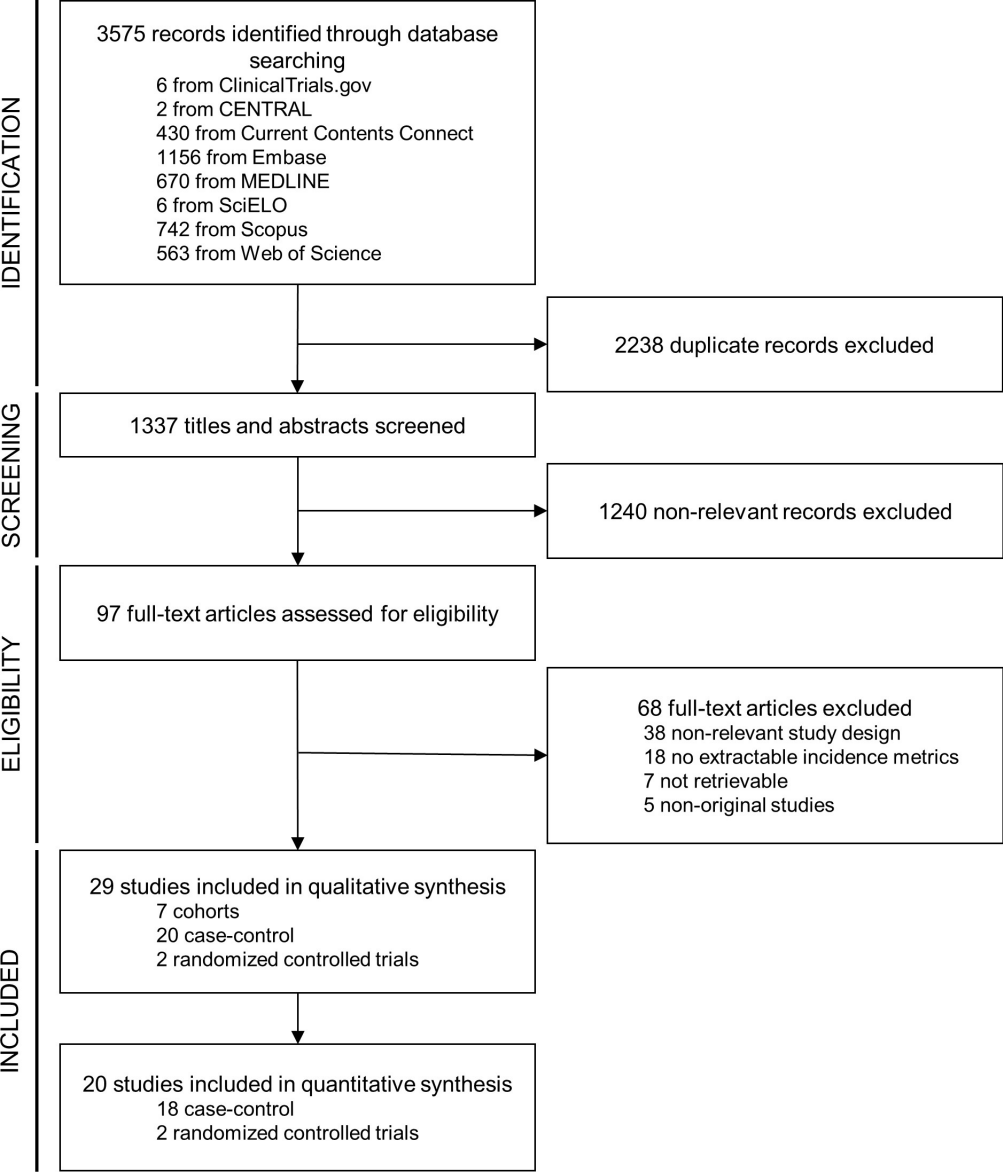
PRISMA flow diagram for study selection process.

The study selection process is illustrated in Fig 1. The initial search yielded a total of 3575 records, of which 2238 were initially removed using the Zotero duplicate detection tool [27], followed by a manual independent screening by JF and NS. During the title and abstract screening, 1240 references were excluded mainly due to: a) being a non-original study or having a non-eligible design; b) not addressing *Mycobacterium ulcerans* infection; c) focusing on other outcomes than the acquisition of BU; d) restricting to specific age groups or forms of the disease. Ninety-seven references were selected for full-text review, at which stage 68 studies were additionally excluded mainly due to not having a compatible study design or providing relevant information for the goal of this review. Contacting of authors was attempted for the retrieval of seven studies, but at the moment of manuscript submission these were still not available.

### Study characteristics and demographic characterization

Demographic characterization of the 29 eligible studies is presented in Table 1. These account for a total of 9265 BU cases, with a generally well-balanced male:female ratio (average of studies: 0.99). Following this line, five of the included sex-unmatched case-control studies [11,16,28–30] (S1 Table), showed comparable sex frequencies between groups (OR = 0.90; 95% CI = 0.71–1.14; *I*^*2*^ = 58%; S1A Fig), independent of restricting analysis to BU laboratory-confirmed cases (OR = 1.01; 95% CI = 0.83–1.24; *I*^*2*^ = 0%; S1B Fig). Regarding age, although data distribution hampers a direct comparison between case-control studies, two of them described children below 15 years of age to be more prevalent amongst cases than controls [10,30] (S2 Table). Remarkably, most cohort studies equally converged on a higher incidence of the disease in this age group, particularly among males, with two additionally reporting another peak of incidence in the elderly [10,31] (Table 1).

**Table 1:** Summary of the included studies.

Study first author [reference]	Study year	Study type	Study period	Country (Continent)	BU cases (n)	Sex (n)		Sex ratio (M:F)	Median age (years)	Age group (years)	n by age group		Sex ratio by age group (M:F)	BU confirmation method (partial/full)	Observations
						Female	Male				Female	Male			
Ahoua L et al. [18]	2009	Case-control	Aug 2001	Ivory Coast (Africa)	116	63	53	0.84	19.5	<15	47		-	AFB, culture, PCR (partial)	Matched by sex, age and village. Reference to BCG vaccination.
										≥15	69				
Aiga H et al. [33]	2004	Case-control	Jul—Aug 1999	Ghana (Africa)	51	21	30	1.43	-	0–14	19	15	0.78	AFB, PCR (partial)	Matched by sex, age group and BCG vaccination.
										15–49	1	11	11		
										≥50	1	4	4		
Bibert S et al. [13]	2017	Case-control	-	Ghana (Africa)	96	57	39	0.68	13	-	-		-	PCR (full)	Matched by sex, age, residence, ethnicity. All cases HIV^-^.
Bratschi MW et al. [32]	2013	Cohort	Mar 2010—Jun 2012	Cameroon (Africa)	157	-	-	-	12.5*	<15	18*	34*	1.89	PCR (partial)	Two peaks in age-adjusted cumulative incidence rates: - 34.4/1 0000 in 12-13-year-old. - 27/1 0000 > 50-year-old.
										15–50	14*	11*	0.79		
										>50	4*	7*	1.75		
Capela C et al. [12]]	2016	Case-control	2005–2015	Benin (Africa)	208	89	119	1.34	14	-	-		-	AFB, culture, PCR (full)	All cases BCG-vaccinated and HIV^-^.
Debacker M et al. [10]	2004	Cohort	1997–2001	Benin (Africa)	1630	882	886	1.00	15	0–14	431	479	1.11	AFB, culture, PCR (partial)	Incidence rates by age and gender, comparing to 15-59-year-old: - Highest risk in > 59-year-old (relative risk (RR) = 3.15, 95% CI = 2.68–3.71, p < 0.001), particularly in males (RR = 1.49, 95% CI = 1.12–1.98, p < 0.006); - Higher risk in 0-14-year-old (RR = 1.19, 95% CI = 1.07–1.32, p = 0.001).
										15–29	226	185	0.82		
										30–44	70	56	0.80		
										45–59	67	65	0.97		
										60–74	75	76	1.01		
										75–89	13	25	1.92		
Debacker M et al. [28]	2006	Case-control	1997–2003	Benin (Africa)	2399	1155	1182	1.02	-	5–9	432		-	-	Unmatched. Reference to BCG vaccination.
										10–14	405				
										15–19	223				
										20–24	163				
										25–29	149				
										30–34	84				
										35–39	72				
										40–44	44				
										45–49	53				
										50–54	68				
										55–59	66				
										60–64	105				
										65–69	65				
										70–74	50				
										75–79	36				
										≥80	17				
Douine M et al. [4]	2017	Cohort	Jan 1969—Dec 2013	French Guiana (South America)	245	121	124	1.02	25	<15	90		-	AFB, culture, histopathology, PCR (partial)	Mean incidences by age varied throughout time: - Before 1984, more pronounced in 5-9-year-old; - After 1999, generally affecting those > 50-year-old.
										≥15	155				
Johnson RC et al. [20]	2008	Case–control	Jan 2002—Aug 2003	Benin (Africa)	426	-	-	-	-	<15	246		-	AFB, culture, PCR (full)	Matched by sex, age and neighborhood. Reference to HIV infection.
										15–49	133				
										≥50	47				
Johnson PDR et al. [34]	2007	Cohort	Jan 2002—Apr 2007	Australia (Oceania)	79	-	-	-	-	0–14	2		-	Culture, PCR (full)	Age-specific attack rate ≈ 7x higher for those > 55-year-old (*p* < 0.001).
										15–24	2				
										25–34	1				
										35–44	4				
										45–54	0				
										55–64	7				
										65–74	9				
										≥74	23				
Kenu E et al. [35]	2014	Case-control	May 2010—Dec 2011	Ghana (Africa)	113	57	56	0.98	28	<10	13		-	AFB, PCR (full)	Matched by sex, age and community. Reference to BCG vaccination.
										11–14	19				
										15–24	19				
										≥24	62				
Landier J et al. [36]	2014	Cohort	2002–2012	Cameroon (Africa)	814	-	-	-	-	-	-		-	Not specified (full)	Incidence rates ratios: - 0.81 (0.67–0.98) for 0-14-year-old; - 1.55 (1.22–1.98) for 15-49-year-old; - 0.85 (0.57–1.28) for > 50-year-old. - 1.01 (95% CI 0.88–1.15) for woman-men. Double incidence rate for 5-14-year-old (165/100 000 person-years) compared with adults (87/100 000 person-years).
Maman I et al. [31]	2018	Case-control	Mar 2013—Mar 2015	Togo (Africa)	83	50	33	0.66	11	<10	39		-	AFB, PCR (full)	Matched by sex and place of residence. Reference to BCG vaccination. All patients HIV-.
										10–14	16				
										15–24	13				
										≥25	15				
Marston BJ et al. [11]	1995	Case-Control	Nov 1991	Ivory Coast (Africa)	70	41	29	0.71	-	<2	0	0	-	AFB (partial)	Matched by region. Rate of illness: - Not statistically different between males (5.2%) and females (7.5%; *p* = 0.11); - Highest among 10-14-year-old (143 cases/1 000; *p* < 0.0005).
										2–4	2	5	2.50		
										5–9	9	6	0.67		
										10–14	10	9	0.90		
										15–19	3	4	1.33		
										20–39	10	4	0.40		
										≥40	6	1	0.17		
N'krumah RTAS et al. [37]	2016	Case-control	Aug—Sep 2012	Ivory Coast (Africa)	51	27	24	0.89	25	<10	4		-	PCR (full)	Matched by sex, age group and living community. Reference to BCG vaccination.
										10–14	9				
										15–25	16				
										26–35	9				
										˃35	13				
Nackers F et al. [38]	2006	Case-Control	Aug 2002—Aug 2003	Benin (Africa)	279	131	148	1.13	13.4	<6	36		-	AFB, culture, histopathology, PCR (partial)	Matched by sex, age and neighborhood. Reference to BCG vaccination, Subanalyses performed with confirmed cases.
										6–12	99				
										13–19	36				
										20–29	39				
										30–39	18				
										≥40	51				
Nackers F et al. [39]	2007	Case-Control	1996—Aug 2003	Benin (Africa)	179	91	88	0.97	-	<6	24		-	-	Matched by sex, age and neighborhood. Reference to genetic variants.
										6–12	56				
										13–19	22				
										20–29	30				
										30–39	16				
										40–59	16				
										≥60	15				
Nackers F et al. [40]	2007	Case-Control	Aug 2002—Aug 2003	Benin (Africa)	324	146	178	1.22	13	-	-		-	AFB, culture, histopathology, PCR (partial)	Matched by sex, age and neighborhood. Reference to BCG vaccination.
O'Brien DP et al. [41]	2017	Cohort	Jan 1998—Apr 2016	Australia (Oceania)	324	160	164	1.03	57	-	-		-	AFB, culture, histopathology, PCR (full)	Incidence of BU among family members (5.69/1 000 persons/year) higher than the estimated for the general population (0.85–4.04/1 000 persons/year).
Phillips RO et al. [29]	2015	Case-control	Feb 2010—Apr 2013	DR Congo (Africa) Ghana (Africa) Togo (Africa)	401	223	178	0.80	13	<9	122		-	AFB, culture, PCR (full)	Reference to BCG vaccination.
										10–19	139				
										20–39	88				
										40–90	52				
Pouillot R et al. [42]	2007	Case-Control	Feb—Mar 2006	Cameroon (Africa)	163 117*	79 61*	84 56*	1.06 0.92*	14 13*	<10	45 31*		-	AFB, PCR (partial)	Matched by age and community or family. Reference to BCG vaccination.
										10–15	41 34*				
										15–24	38 27*				
										≥24	39 25*				
Quek TYJ et al. [30]	2007	Case-control	1998–2005	Australia (Oceania)	49	25	24	0.96	70	≤60	16		-	Culture, PCR (full)	Unmatched. Excluded patients < 20 years of age. Reference to BCG vaccination.
										>60	33				
Raghunathan PL et al. [16]	2005	Case-control	Aug—Nov 2000	Ghana (Africa)	121	64	57	0.89	12	<15	72		-	AFB, culture. histopathology, PCR (full)	Matched by age and village. Reference to BCG vaccination and HIV infection.
										≥15	49				
Smith PG et al. [43]	1976	RCT	Jul—Sep 1970	Uganda (Africa)	602	317	285	0.90		0–4	37	37	1	Culture, histopathology (partial)	Reference to BCG vaccination.
										5–9	73	93	1.27		
										10–14	78	86	1.10		
										15–19	46	33	0.72		
										20–24	19	12	0.63		
										25+	64	24	0.38		
Sopoh GE et al. [15]	2010	Case-control	Jan 2006—Jun 2008	Benin (Africa)	104	43	61	1.42	12	<15	62		-	AFB, culture, PCR (full)	Matched by sex, age and village.
										≥15	42				
Stienstra Y et al. [44]	2004	Case-control	Sep—Nov 2000	Ghana (Africa)	106	54	52	0.96	12	-	-		-	AFB, culture, histopathology, PCR (full)	Matched by age and community. Reference to HIV infection.
Stienstra Y et al. [14]	2006	Case-control	2000, 2003	Ghana (Africa)	182	-	-	-	13	-	-		-	AFB, culture, histopathology, PCR (partial)	Matched by neighborhood.
Uganda Buruli Group [45]	1969	RCT	1967	Uganda (Africa)	127	66	61	0.92	-	1–2	3	3	1.00	-	Fall in the protection-rate when comparing the first 6 months post-vaccination with the following 6 months (*p* = 0.047).
										3–4	7	5	0.71		
										5–9	12	21	1.75		
										10–14	16	21	1.31		
										15–19	10	6	0.60		
										20–24	5	2	0.40		
										≥25	13	3	0.23		
Uganda Buruli Group [46]	1971	Case-Control	Jan 1966—Jan 1970	Uganda (Africa)	220	110	110	1.00	-	0	2	3	1.50	-	Reference to BCG vaccination.
										1–2	11	3	0.27		
										3–4	8	9	1.13		
										5–9	35	22	0.63		
										10–14	32	24	0.75		
										15–19	11	16	1.45		
										20–24	4	7	1.75		
										≥25	7	26	3.71		

*Subset of BU patients with laboratory-confirmed BU disease.

### Family history of BU

One cohort study found higher rates of BU in family members of BU patients (5.69/1000 person-years) when comparing the estimated rates for the general population of the region (0.85–4.04 cases/year/1000 population; [41] (Table 1). Among the five case-control studies registering this variable, only one adjusted the analyses for non-consanguineous bonds [15], while two included household relationships [33,40] and the other two did not provide any description on the variable [16,18] (S3 Table). Pooling of available data from studies comprising probable (OR = 1.14; 95% CI = 0.70–1.88; *I*^*2*^ = 54%; S2A Fig) or laboratory-confirmed (OR = 1.01; 95% CI = 0.61–1.68; *I*^*2*^ = 54%; S2B Fig) cases revealed no significant impact of family history in BU development, although a moderate heterogeneity is noted.

### Genetic variants

**Fig 2:**
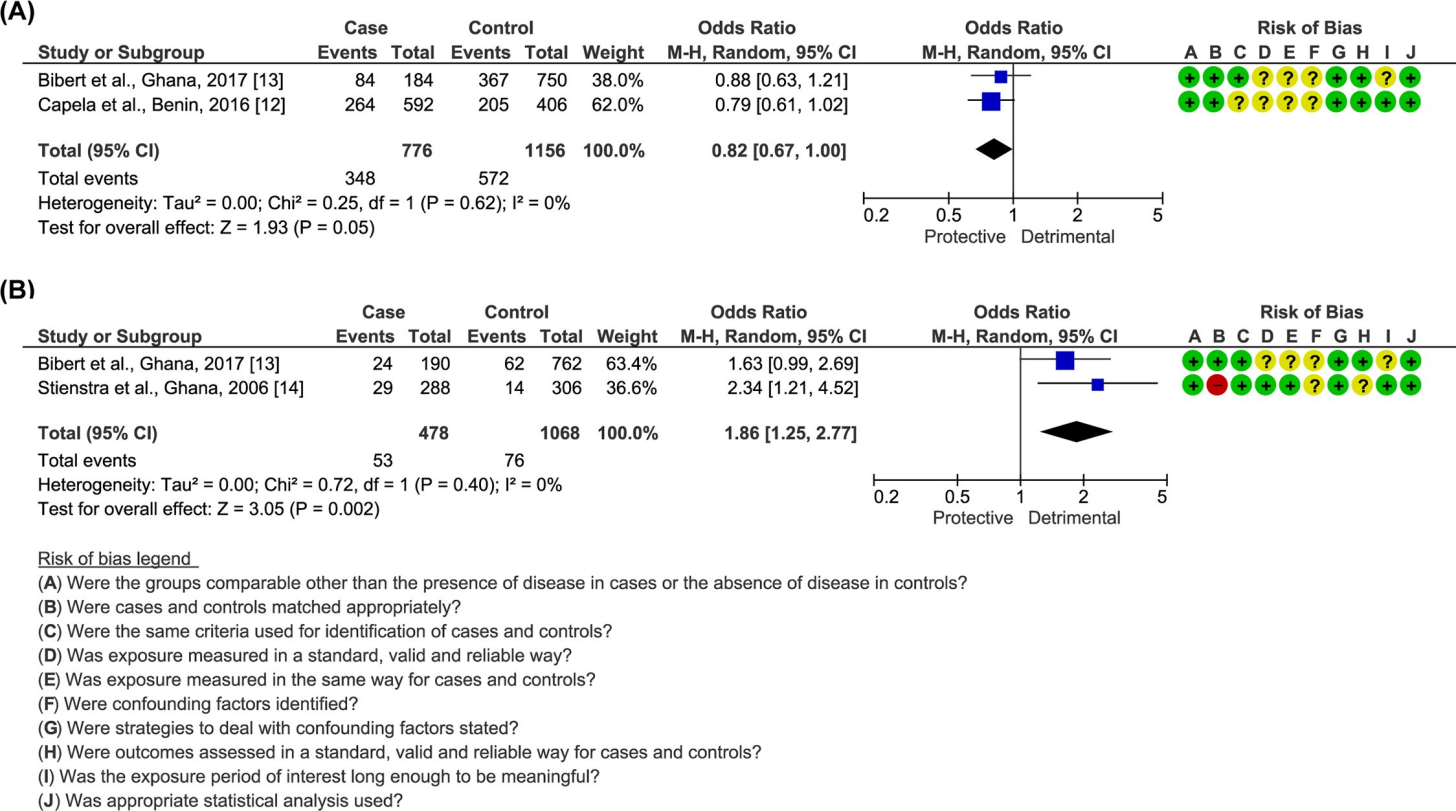
Comparison of the frequency of allelic variants among BU patients and endemic controls. The number of events indicates the frequency of the minor allele. (A) *PARK2* rs1040079. (B) *SLC11A1* rs17235409. Bias: **+** low risk;—high risk; **?** unclear risk.

Looking at the four genetic association studies captured by this systematic review [12–14,39], it is foremost noticeable a discrepancy in the statistical approaches used by the different authors, hampering direct comparison and interpretation of results (S4 Table). As such, allelic frequencies were calculated from the available data, demonstrating minor alleles in *iNOS* rs9282799 (OR = 2.11; 95% CI = 1.27–3.50), *IFNG* rs2069705 (OR = 1.55; 95% CI = 1.13–2.14), *PARK2* rs1333955 (OR = 1.36; 95% CI = 1.01–1.82) and *SLC11A1* rs17235409 (OR = 2.34; 95% CI = 1.21–4.52) to be more likely present in BU patients than in controls, while the opposite was true in the case of *IFNG* rs3138557 (OR = 0.59; 95% CI = 0.40–0.85; S4 Table). Additionally, two SNPs *PARK2* rs1040079 and *SLC11A1* rs17235409 were studied in more than one setting and thus had their effects weighed (S4 Table). Overall, *PARK2* rs1040079 showed a small to no significant association with BU prevalence (OR = 0.82; 95% CI = 0.67–1.00, *I*^*2*^ *=* 0%; Fig 2A), apparently more relevant when considering a recessive model of inheritance (OR = 0.68; 95% CI = 0.48–0.98; *I*^*2*^ *=* 0%; S3 Fig). As for *SLC11A1* rs17235409, aggregation of studies resulted in a detrimental role for the minor allele (OR = 1.86; 95% CI = 1.25–2.77; *I*^*2*^ = 0%; Fig 2B), namely when modelled in dominance (OR = 2.10; 95% CI = 1.37–3.21; *I*^*2*^ = 0%) or overdominance (OR = 2.21; 95% CI = 1.45–3.39; *I*^*2*^ = 0%; S4 Fig).

### BCG vaccination

**Fig 3:**
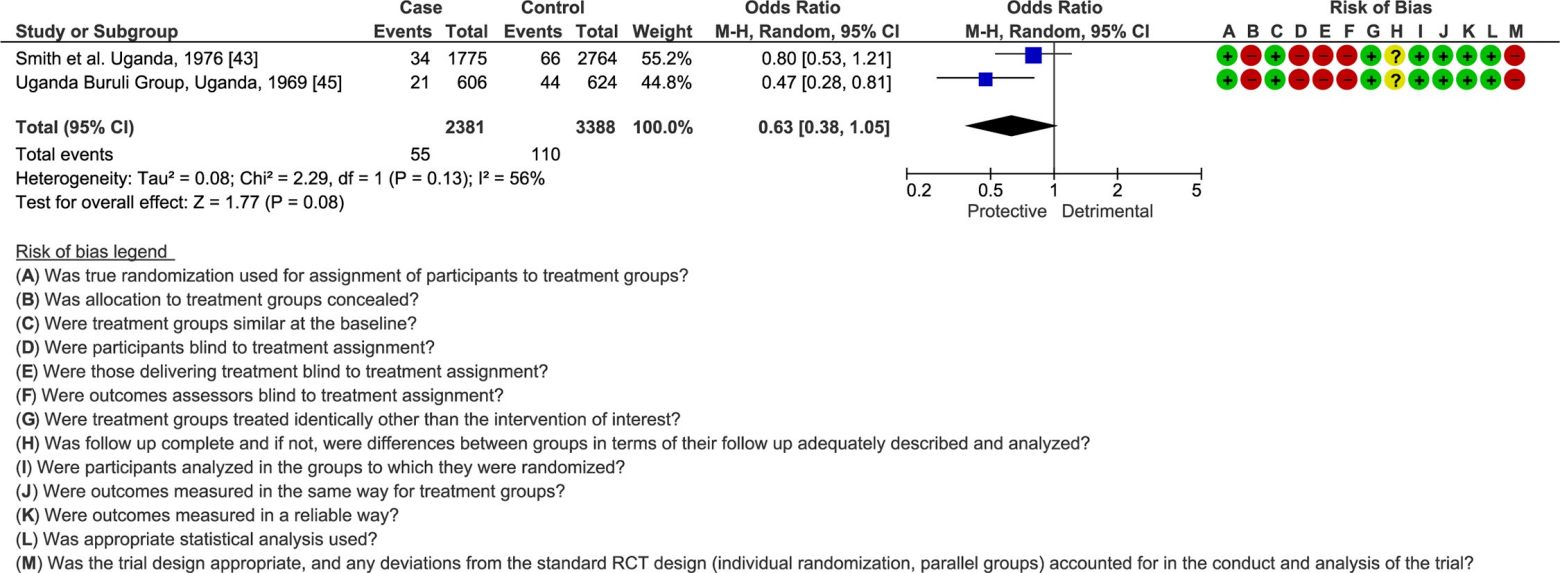
Comparison of the frequency of BU among BCG vaccinated and non-vaccinated individuals in RCTs. The number of events indicates BCG**-**vaccinated individuals. Bias: **+** low risk;—high risk; **?** unclear risk.

Only two RCTs attempted to scrutinize the role of BCG in protecting against BU [43,45], both concluding on a short-term positive effect for the vaccine (S5 Table). However, studies were moderately heterogeneous, demonstrating a negligible effect for the vaccine when analyzed under a random-effects model (OR = 0.63; 95% CI = 0.38–1.05; *I*^*2*^ = 56%; Fig 3).

**Fig 4:**
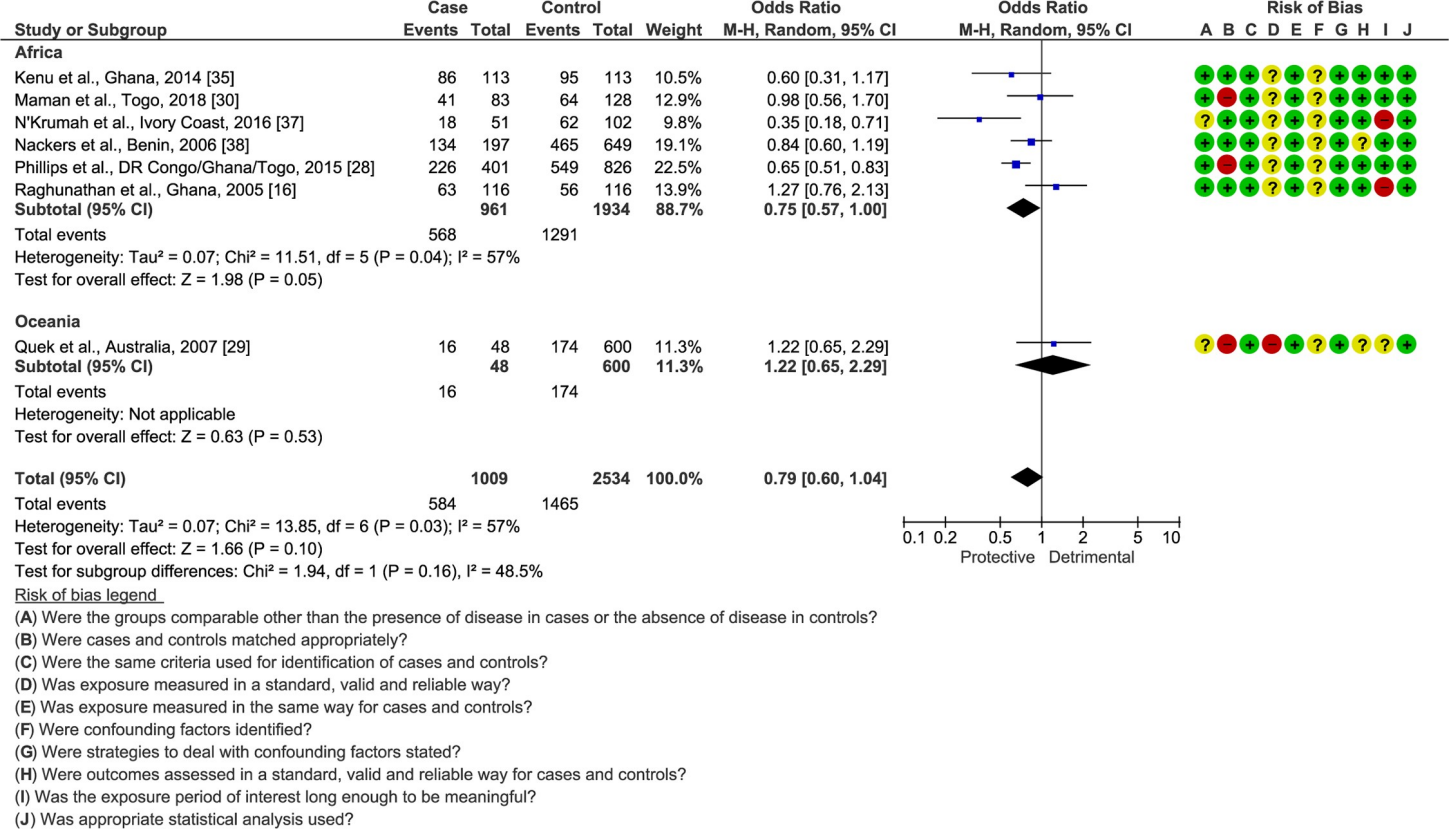
Comparison of the frequency of BCG vaccination among laboratory-confirmed BU patients and endemic controls in case-control studies. The number of events indicates BCG**-**vaccinated individuals. Bias: **+** low risk;—high risk; **?** unclear risk.

Ten case-control studies determined BCG-vaccination status, mostly through BCG scar, although one did report the vaccine strains under use at the time of evaluation [28] (S5 Table). Results among authors were highly heterogeneous (Tau^2^ = 0.48; Chi^2^ = 126.03; *p* < 0.00001; *I*^*2*^ = 93%), with four studies showing a protective [18,29,37,42] (total n participants = 1953), five a neutral [16,30,31,35,38] (total n participants = 2338), and one a detrimental [10] (total n participants = 2742) role for the vaccine (S5 Fig). Thus, focus was placed exclusively on results derived from laboratory-confirmed cases, which despite still heterogenous (Tau^2^ = 0.07; Chi^2^ = 13.85; *p* = 0.03; *I*^*2*^ = 57%), suggested that the overall OR of BU acquisition is similar between vaccinated and unvaccinated individuals (OR = 0.79; 95% CI = 0.60–1.04; Fig 4).

### HIV infection/co-infection

**Fig 5:**
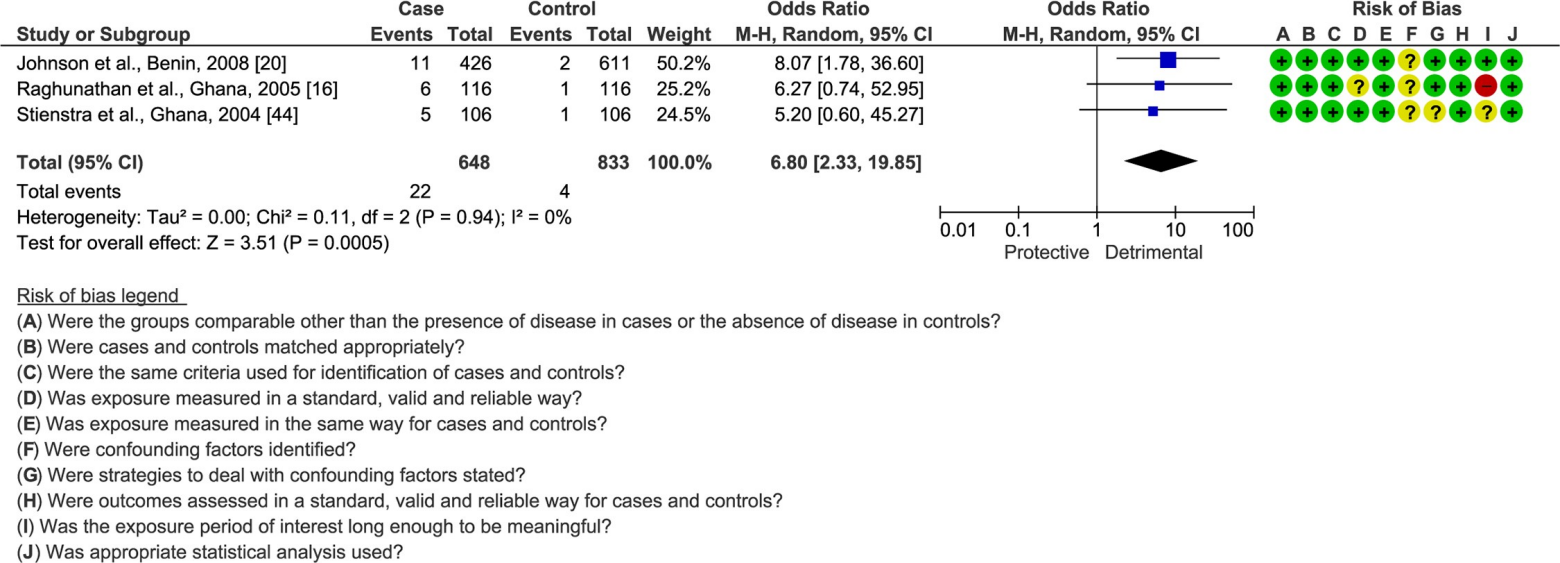
Comparison of frequency of HIV infection among BU patients and endemic controls. The number of events indicates HIV^**+**^ individuals. Bias: **+** low risk;—high risk; **?** unclear risk.

Three studies evaluated HIV serotype 1/2 status of case and control participants [16,20,44] (S6 Table). While the number of HIV patients was small, they were homogeneous in showing HIV infection being associated with higher BU prevalence (OR = 6.80; 95% CI = 2.33–19.85; *I*^*2*^ = 0%; *I*^*2*^ = 0%; Fig 5), regardless of the confirmation of BU diagnosis (OR = 6.40; 95% CI = 2.12–19.29; *I*^*2*^ = 0%; S6 Fig).

### Risk of bias

The summary of the risk of bias among studies can be found in S7–S9 Tables. Unclear effects of selection and detection biases should be considered when analyzing all studies, mainly due to the lack of incorporation of the social history and BCG vaccination and HIV infection statuses of BU patients, or the use of the unreliable BCG scar as a source of vaccination status check [47]. Considering a mean incubation period of *M*. *ulcerans* of 4.8 months, it is also impossible to warrant that the studies reviewed only included individuals free-of-outcome *ad initium* [48]. This timeframe was also considered in the evaluation of exposure periods, which was here established as minimum for the correct sorting of controls. Furthermore, literature supports that BU clinical diagnosis can be correctly achieved in over 90% of cases, although this value is dependent on the experience of the physician documenting the event [49]. This reinforces the assertions made by WHO and other authors to confirm BU cases by any of the specified laboratorial methods [2,49]. Finally, there appeared to be a reporting bias in the BCG analysis, as observed by the asymmetric funnel plot of the studies (S7 Fig).

## Discussion

Demographic variables of BU patients have been reviewed by many authors, with no strict consensus having been achieved as to their role as a risk modifier for disease acquisition [7,50]. Results here obtained point to a bipartisan role of age and sex in the susceptibility to BU, as the disease is more frequently observed among children, particularly males, and in the elderly. Interestingly, these patterns can also be observed in other studies not fulfilling the inclusion criteria for this systematic review (S10 Table). In younger generations, it has been previously argued that these differences could be explained based on increased exposure to *M*. *ulcerans*, mainly due to the more erratic behavior that makes children more prone to skin lesions, as well as their likelihood to stay near aquatic environments [50]. However, more recent data encompassing worse outcomes of BU among the elderly casted a stronger suspicion on age as a modulator of intrinsic processes, likely immunological, a phenomenon that could be transversal across generations [41,51]. As such, while age and sex have the ability to influence the disease process, it is still uncertain at this point why.

Suspicion on a role for inheritance in susceptibility to BU can be traced to a case series from 1990, fostered by the question: why, in a community of individuals with similar environmental exposure, do some develop the disease while others do not [15,52]? Unfortunately, not all studies have used the same criteria to assess this variable, significantly increasing the chance of incorporating the confounding effects of environmental exposure (S6 Table). In this regard, when considering exclusively non-consanguine relationships, Sopoh et al. found the odds of acquiring BU to increase the susceptibility to the disease (adjusted OR = 5.5; 95% CI = 3.0–10.0), which altogether with previous evidence suggests BU resistance/susceptibility can be inherited [15,17,41].

Genetic association studies performed so far also point to a role for genetics in BU. More importantly, not only does the study of SNPs help define further risk factors for BU development, but it also reinforces findings from cellular and molecular research, as seen, for instance, with interferon-gamma [13,53]. From the SNPs that were addressed in more than one study, data revealed that the minor allele from *SLC11A1* rs17235409 was associated with decreased prevalence of the disease. Besides its role as a divalent cation transport, SLC11A1 (solute carrier family 11, member 1) possesses several other immunomodulatory properties, having also been significantly associated with other mycobacterioses in another meta-analysis [54,55]. Likewise, parkin, encoded by *PARK2*, can mediate an antimycobacterial response through protein ubiquitination, a process critical for autophagic targeting of intracellular pathogens [56]. Despite the absence of effect estimated for *PARK2* rs1040079 in BU acquisition, Capela et al. found that the allelic variant in *PARK2* rs1333955 conferred susceptibility to the disease, when modelling for dominant model of genetic inheritance [12]. As of now, these SNPs could be considered for population screening and stratification. However, in the future, larger scale genomic studies may point to new variants that open avenues for novel therapeutic targets.

The WHO recently advocated BCG vaccination in BU endemic countries, based on the results of a systematic review and meta-analysis from Zimmermann et al. demonstrating a protective effect for BCG, at least when considering the RCTs included in the study [8,19]. By analyzing data of RCTs and case-control studies within a random-effects modelling, the present study suggests that BCG does not have a meaningful impact in protecting against BU. In fact, and similar to what was already observed in tuberculosis, the protection conferred by the vaccine appears to wane over time, being in BU apparently restricted to the first 6 months post-vaccination [43,57]. Moreover, from the 4-year follow-up made by Smith et al. it is possible to estimate that it is necessary to vaccinate at least 125 individuals in order to prevent one case of BU within this timeframe [43,45]. Still, whether stratification of these results by BCG strains could have exposed different results remains an interrogation, as this is a factor known to influence immune cellular response [58]. Thus, based on current evidence, the usefulness of BCG in BU appears to be essentially restricted to protecting against osteomyelitis forms of the disease [59,60].

Contrary to tuberculosis, in which HIV coinfection is well-known to foster active infection, evidence for such role in BU has been scarcer [7,61]. The studies gathered in this systematic review allow to draw a similar conclusion regarding BU development, further supported by estimates of a generally higher HIV prevalence among BU patients [21,62]. Indeed, not only does HIV favor the acquisition of BU, but Christinet et al. also described increased lesion size and severity, together with more prolonged wound healing periods in BU HIV^+^ patients with low CD4 T cell counts [62]. Thus, immune compromise by HIV infection hampers control of *M*. *ulcerans*, findings that not only have consequences at the populational level but may prove helpful to advance the understanding of the disease mechanisms.

This study represents the first meta-analysis on clinical risk factors for BU beyond BCG vaccination. Its major strengths rely on the broad search criteria employed, inclusion and ponderation of results from a large number of variables from both RCTs and observational studies, as well as the consideration of the BU-confirmation status of cases in the analyses. In contrast, it is limited by the level of evidence provided by the studies, the actual number of studies that addressed some of the risk factors here reviewed, as well by a lack of uniformity among definitions and study designs seen across the literature. Thus, some recommendations for more robust epidemiological studies are here proposed: consideration of the confirmation-status of BU patients throughout analyses; reporting and/or more thorough evaluation of the exposure variables here addressed; and the use of consistent cut-off values, namely for age groups, to allow data comparison.

Overall, this work contributes with the notion that there is a bimodal peak of incidence in BU, namely among children and older adults, although the underlying causes are yet to be unraveled. Furthermore, evidence suggests BCG vaccination is not suitable to prevent BU in the mid-long term, prompting even further for the investigation of better vaccination alternatives. Additionally, this review found a higher prevalence of the disease in HIV-infected individuals and also carriers of genetic polymorphisms in *SLC11A1*, both of which can be easily detected by low-cost methods and thus be used for BU risk stratification. Altogether, these findings contribute to the understanding of BU and should be regarded when advocating for new global health policies against this neglected tropical disease.

## Supporting information

S1 Checklist PRISMA checklist. (PDF)

S1 Text Search strategy. (PDF)

S1 Table Sex-related comparisons in sex unmatched case-control studies. (PDF)

S2 Table Age-related comparisons in age unmatched case-control studies. (PDF)

S3 Table Family history of BU-related comparisons in case-control studies. (PDF)

S4 Table Allelic frequency-related comparisons in genetic association studies. (PDF)

S5 Table BCG vaccination-related comparisons in randomized clinical trials (RCT) and case-control studies. (PDF)

S6 Table Human Immunodeficiency Virus (HIV) infection-related comparisons in case-control studies. (PDF)

S7 Table Risk of bias among the cohort studies included in the systematic review. (PDF)

S8 Table Risk of bias among the case-control studies included in meta-analysis. (PDF)

S9 Table Risk of bias among the RCTs included in meta-analysis. (PDF)

S10 Table Demographic information from relevant observational and case series studies not eligible for systematic review. Overall male:female ratio = 1.06.
(PDF)

S1 Fig Comparison of the frequency of sexes among BU patients and endemic controls in sex unmatched case-control studies. The number of events indicates female individuals. (A) All BU cases included. (B) BU laboratory-confirmed cases only. Bias: + low risk;—high risk; ? unclear risk.
(TIF)

S2 Fig Comparison of the frequency of family history of BU among BU patients and endemic controls. The number of events indicates patients with BU history in family members. (A) All BU cases included. (B) BU laboratory-confirmed cases only. Bias: + low risk;—high risk; ? unclear risk.
(TIF)

S3 Fig Comparison of the frequency of PARK2 rs1040079 polymorphism among BU patients and endemic controls according to different genetic models of inheritance. (A) Dominant. (B) Overdominant. (C) Recessive.
(TIF)

S4 Fig Comparison of the frequency of SLC11A1 rs17235409 polymorphism among BU patients and endemic controls according to different genetic models of inheritance. (A) Dominant. (B) Overdominant.
(TIF)

S5 Fig Comparison of the frequency of BCG vaccination among BU patients and endemic controls in all of the included case-control studies. The number of events indicates BCG-vaccinated individuals. Bias: + low risk;—high risk; ? unclear risk.
(TIF)

S6 Fig Comparison of the frequency of HIV infection among BU laboratory-confirmed patients and endemic controls. Bias: + low risk;—high risk; ? unclear risk.
(TIF)

S7 Fig Funnel plot of BCG vaccination studies. Each symbol is representative of a reference. SE–standard error.
(TIF)
