# Supplementary material for: Individual and clinical variables associated with the risk of Buruli ulcer acquisition: A systematic review and meta-analysis
Source: PLoS Negl Trop Dis. 2020 Apr 8;14(4):e0008161. doi: 10.1371/journal.pntd.0008161 (PMC7170268; doi:10.1371/journal.pntd.0008161)
Supplement: S1 Text — (PDF) [file pntd.0008161.s002.pdf]

S1 Text. Search Strategy.
Below is the search strategy employed in MEDLINE to identify potentially relevant studies. Medical Subject Heading (MESH) terms are exploded and are marked in bold.

1. buruli OR bairnsdale OR daintree
2. ulcer
3. 1 AND 2
4. mycobacterium
5. ulcerans
6. 4 AND 5
7. 3 OR 6
8. Epidemiol* OR prevalence OR incidence OR susceptibility OR transmission OR odd OR risk OR **epidemiologic studies** OR **clinical studies as topic**
9. 7 AND 8

Below is the search strategy employed in Embase to identify potentially relevant studies. Emtree terms are exploded and are marked in bold.

1. buruli OR bairnsdale OR daintree
2. ulcer
3. 1 AND 2
4. mycobacterium
5. ulcerans
6. 4 AND 5
7. 3 OR 6
8. Epidemiol* OR prevalence OR incidence OR susceptibility OR transmission OR odd OR risk OR **epidemiology** OR **epidemiological data** OR **disease predisposition** OR **disease transmission** OR **risk** OR **clinical study**
9. 7 AND 8

Below is the search strategy employed in Current Contents Connect, Scopus and Web of Science to identify potentially relevant studies:

1. buruli OR bairnsdale OR daintree
2. ulcer
3. 1 AND 2
4. mycobacterium
5. ulcerans
6. 4 AND 5
7. 3 OR 6
8. Epidemiol* OR prevalence OR incidence OR susceptibility OR transmission OR odd OR risk OR observational OR case-control OR cohort OR OR trial
9. 7 AND 8

Below is the search strategy employed in SciELO to identify potentially relevant studies:

1. buruli OR bairnsdale OR daintree
2. ulcer OR úlcera
3. 1 AND 2
4. mycobacterium
5. ulcerans
6. 4 AND 5
7. 3 OR 6

Below is the search strategy employed in Cochrane Controlled Register of Trials (CENTRAL) to identify potentially relevant studies:
1. buruli OR bairnsdale OR daintree
2. ulcer
3. 1 AND 2
4. mycobacterium

5. ulcerans
6. 4 AND 5
7. 3 OR 6
8. BCG OR vaccine*
9. 7 AND 8

Below is the search strategy employed in ClinicalTrials.gov to identify potentially relevant studies:

1. buruli OR bairnsdale OR daintree
2. ulcer
3. 1 AND 2
