# Supplementary material for: Individual and clinical variables associated with the risk of Buruli ulcer acquisition: A systematic review and meta-analysis
Source: PLoS Negl Trop Dis. 2020 Apr 8;14(4):e0008161. doi: 10.1371/journal.pntd.0008161 (PMC7170268; doi:10.1371/journal.pntd.0008161)
Supplement: S1 Table — (PDF) [file pntd.0008161.s003.pdf]

S1 Table. Sex-related comparisons in sex unmatched case-control studies.

Study first author [reference]	Female sex cases (%)	Female sex controls (%)	*p value*	Crude OR	Adjusted OR	Confounders included in adjusted estimates	Observations
Debacker M et al. [33]	1115 (47.7)	760 (54.8)	<0.01*	0.78 (0.68-0.89)*	For school-aged children:<br>- 4.63 (95% CI 2.63–8.15) in boys.<br>- 2.75 (95% CI 1.59–4.76) in girls.	Age, region, BCG vaccination scar, water sources	Children < 5 years of age sampled from a different clinic.<br>Male OR for BU:<br>- 0.85 (95% CI = 0.59–1.22) in < 5-year-old;<br>- 1.36 (95% CI = 1.18—1.47) in > 5-year-old.
Marston BJ et al. [11]	26 (56.5)	45 (50)	-	1.30	-	Age, residence in encampment, washing clothes, rice farming, mean number of days in rice field, mean time to walk to the Lobo river from farm rice, corn farming, wearing long pants	No association found in multivariate analysis.
Phillips RO et al. [28]	223 (55.61)	445 (53.87)	0.57	1.07 (0.84-1.36)*	-	-	-
Pouillot R et al. [42]	79 (48)	-	> 0.05 in comparisons with family-matched controls	-	-	-	No demographics information provided on controls.
Quek TYJ et al. [29]	25 (51)	324 (54)	0.90	0.93 (0.51-1.67)*	-	-	-
Raghunathan PL et al. [16]	62 (53)	67 (49)	0.25	0.84 (0.50, 1.41])*	-	-	-
Stienstra Y et al. [14]	-	-	0.583	-	-	-	-
Stienstra Y et al. [44]	-	-	-	-	-	-	No information provided.

*Calculated from the available data.
