# Supplementary material for: Individual and clinical variables associated with the risk of Buruli ulcer acquisition: A systematic review and meta-analysis
Source: PLoS Negl Trop Dis. 2020 Apr 8;14(4):e0008161. doi: 10.1371/journal.pntd.0008161 (PMC7170268; doi:10.1371/journal.pntd.0008161)
Supplement: S2 Table — (PDF) [file pntd.0008161.s004.pdf]

S2 Table. Age-related comparisons in age unmatched case-control studies.

Study first author [reference]	Mean age cases (±SD)	Mean age cases (±SD)	Median age cases (IQR)	Median age controls (IQR)	*p* value	Confounders included in adjusted estimates	Observations
Debacker M et al. [33]	-	-	-	-	-	Age, region, BCG vaccination scar, water sources	Metrics provided only for age groups. Adjusted OR for BU: - 6.74 (95% CI 2.67–17.03) in 3-4-year-old; - 11.64 (95% CI 8.01–16.91) in 5-14-year-old; - 4.36 (95% CI 3.04–6.27) in > 50-year-old.
Maman I et al. [30]	-	-	11 (3-65)*	19 (8-60)*	0.001	Age, receiving insect bites near a river, bathing with water from open borehole	Metrics provided only for age groups. Adjusted OR for BU: - 11.48 (95% CI = 3.72–35.43) < 10-year-old; - 3.63 (95% CI = 1.22–10.83) in 10-14-year-old.
Marston BJ et al. [11]	16.0	21.4	-	-	0.04	Age, residence in encampment, washing clothes, rice farming, mean number of days in rice field, mean time to walk to the Lobo river from farm rice, corn farming, wearing long pants	No association found in multivariate analysis.
Phillips RO et al. [28]	-	-	13 (8-27)	16 (9-30)	0.01	Country	Additional metrics provided for age groups.
Pouillot R et al. [42]	-	-	14 (1-71)	-	> 0.05 in comparisons with family-matched controls	-	No demographics information provided on controls.
Quek TYJ et al. [29]	-	-	70 (58-82)	61 (48-72)	0.01	-	-
Stienstra Y et al. [44]	-	-	13 (8-21)	13 (9-22)	-	-	-

*Range reported instead of interquartile range (IQR).
