# Supplementary material for: Individual and clinical variables associated with the risk of Buruli ulcer acquisition: A systematic review and meta-analysis
Source: PLoS Negl Trop Dis. 2020 Apr 8;14(4):e0008161. doi: 10.1371/journal.pntd.0008161 (PMC7170268; doi:10.1371/journal.pntd.0008161)
Supplement: S3 Table — (PDF) [file pntd.0008161.s005.pdf]

S3 Table. Family history of BU-related comparisons in case-control studies.

Study first author [reference]	Cases with family history of BU (%)	Controls with family history of BU (%)	Crude OR (95% CI)	Adjusted OR (95% CI)	Confounders included in adjusted estimates	Observations
Ahoua L et al. [18]	45 (70)	39 (56)	1.8 (0.8-4.0)	-	-	-
Aiga H et al. [32]	12 (23.5)	6 (11.8)	2.20 (0.90-7.25)	-	-	Household members included.
Nackers F et al. [40]	38 (12.8)	127 (12.7)	1.05 (0.70-1.60)	-	-	Wife/husband relationships included.
Raghunathan PL et al. [16]	14 (12)	22 (19)	0.58 (0.28–1.22)	-	-	-
Sopoh GE et al. [15]	-	-	5.07 (2.81–9.14)	5.5 (3.0–10.0)	Age, marital status, hereditary disease in the family	Only consanguineous relationships.
