# Supplementary material for: Individual and clinical variables associated with the risk of Buruli ulcer acquisition: A systematic review and meta-analysis
Source: PLoS Negl Trop Dis. 2020 Apr 8;14(4):e0008161. doi: 10.1371/journal.pntd.0008161 (PMC7170268; doi:10.1371/journal.pntd.0008161)
Supplement: S4 Table — (PDF) [file pntd.0008161.s006.pdf]

S4 Table. Allelic frequency-related comparisons in genetic association studies.

Study first author [reference]	Genotyping method	Statistical approach	Gene	SNP rs# number	Genotype	Cases (%)	Controls (%)	Minor allele cases (n total)	Minor allele controls (n total)	Calculated allelic OR (95% CI)
Bibert S et al. [13]	GoldenGate SNP Assay	Logistic regression, assuming additive model of inheritance	*iNOS*	rs9282799	GG, GA, AA	71 (75.5), 21 (22.3), 2 (2.1)	329 (87.5), 43 (11.4), 4 (1.1)	25 (188)	51 (752)	**2.11 (1.27-3.50)**
				rs8078340	GG, GA, AA	70 (73.7), 21 (22.1), 4 (4.2)	264 (66.3), 118 (29.6), 16 (4.0)	29 (190)	150 (796)	0.78 (0.50-1.20)
			IFNG	rs2069705	AA, AG, GG	18 (18.9), 48 (50.5), 29 (30.5)	113 (29.9), 191 (50.5), 74 (19.6)	106 (190)	339 (756)	**1.55 (1.13-2.14)**
				rs3138557	(CA)14/15	57 (60.0), 34 (35.8), 4 (4.2)	180 (47.1), 155 (40.6), 47 (12.3)	42 (190)	249 (764)	**0.59 (0.40-0.85)**
			SLC11A1	rs17235409	GG, GA, AA	71 (74.7), 24 (25.3), 0 (0.0)	323 (84.8), 54 (14.2), 4 (1.0)	24 (190)	62 (762)	1.63 (0.99-2.69)
			PARK2	rs1040079	AA, AG, GG	21 (22.8), 58 (63.0), 13 (14.1)	96 (25.6), 191 (50.9), 88 (23.5)	84 (184)	367 (750)	0.88 (0.63-1.21)
			VDR	rs731236	AA, AG, GG	61 (64.2), 30 (31.6), 4 (4.2)	229 (60.3), 126 (33.2), 25 (6.6)	38 (190)	176 (760)	0.83 (0.56-1.23)
				rs7975232	AA, AC, CC	38 (44.2), 39 (45.3), 9 (10.5)	191 (55.2), 122 (35.3), 33 (9.5)	57 (172)	188 (692)	1.33 (0.93-1.90)
			NOD2	rs9302752	GG, GA, AA	36 (37.9), 43 (45.3), 16 (16.8)	148 (39.2), 174 (46.0), 56 (14.8)	75 (190)	286 (756)	1.07 (0.77-1.48)
				rs7194886	GG, GA, AA	56 (58.9), 29 (30.5), 10 (10.5)	196 (51.7), 154 (40.6), 29 (7.7)	49 (190)	212 (758)	0.90 (0.62-1.28)
Capela C et al. [12]	KASP genotyping chemistry	Fisher exact test considering allelic frequencies and different models of inheritance	*PARK2*	rs1333955	TT, TC, CC	107 (52.2), 83 (40.5), 15 (7.3)	177 (61.0), 99 (34.1), 14 (4.8)	113 (410)	127 (580)	**1.36 (1.01-1.82)**
				rs1040079	AA, AG, GG	90 (30.4), 148 (50.0), 58 (19.6)	47 (23.2), 107 (52.7), 49 (24.1)	264 (592)	205 (406)	0.79 (0.61-1.02)
				rs1514343	GG, GA, AA	70 (33.7), 109 (52.4), 29 (13.9)	97 (32.4), 145 (48.5), 57 (19.1)	167 (416)	259 (598)	0.88 (0.68-1.13)
Nackers F et al. [39]	TaqMan MGB® (minor groove binder)	Pearson $\chi 2$ test considering combinations of hemoglobin A, S and C genotypes	*HBB*	rs334	AA, AT, TT	125 (79.1), 32 (20.3), 1 (0.6)	176 (80.7), 41 (18.8), 1 (0.5)	34 (316)	43 (436)	1.10 (0.69-1.77)
				rs33930165	CC, CT, TT	125 (86.2), 19 (13.1), 1 (0.7)	176 (88.4), 20 (10.1), 3 (1.5)	21 (290)	26 (398)	1.12 (0.62-2.03)
Stienstra Y et al. [14]	TaqMan MGB® (minor groove binder)	Pearson $\chi 2$ test or Fisher's exact test comparing to the most common genotype	*SLC11A1*	rs3731685	GG, GC, CC	150 (88.8), 19 (11.2), 0 (0.0)	167 (90.8), 16 (8.7), 1 (0.5)	19 (338)	18 (368)	1.16 (0.60-2.25)
				rs17235409	GG, GA, AA	115 (79.9), 29 (20.1), 0 (0.0)	139 (90.8), 14 (9.2), 0 (0.0)	29 (288)	14 (306)	**2.34 (1.21-4.52)**
				rs17235416	TGTG ins/ins, ins/del, del/del	81 (54.0), 62 (41.3), 7 (4.7)	96 (55.2), 64 (36.8), 14 (8.0)	76 (300)	92 (348)	0.94 (0.66-1.34)
