# Supplementary material for: Individual and clinical variables associated with the risk of Buruli ulcer acquisition: A systematic review and meta-analysis
Source: PLoS Negl Trop Dis. 2020 Apr 8;14(4):e0008161. doi: 10.1371/journal.pntd.0008161 (PMC7170268; doi:10.1371/journal.pntd.0008161)
Supplement: S5 Table — (PDF) [file pntd.0008161.s007.pdf]

S5 Table. BCG vaccination-related comparisons in randomized clinical trials (RCT) and case-control studies.

Study first author [reference]	Study type	BCG determination method	BCG vaccine origin	BCG-vaccinated cases (%)	BCG-vaccinated controls (%)	Crude OR (95% CI)	Adjusted OR (95% CI)	Confounders included in adjusted estimates	Observations
Ahoua L et al. [18]	Case-control	Questionnaire	-	62 (53)	88 (76)	2.4 (1.4 - 4.3)*	5.0 (1.7-14.3)*	Age, type of water point near the housing, place for fishing, sex and region of residency	-
Debacker M et al. [33]	Case-control	Scar	-	158 (92.4), <5 years of age	170 (97.1), <5 years of age	0.35 (0.12-1.02)*	-	-	-
				969 (75.6), ≥5 years of age	610 (55.7), ≥5 years of age	2.43 (2.08-2.94)*	2.5 (1.92-3.22)*	Age, region and water sources	
Kenu E et al. [35]	Case-control	Scar	-	86 (76.1)	95 (84.1)	0.60 (0.31-1.20)	-	-	-
Maman I et al. [30]	Case-control	Scar	-	41 (51.2)	64 (50.4)	1.03 (0.59-1.18)	-	-	-
N'krumah RTAS et al. [37]	Case-control	Questionnaire/ Scar	-	18 (35.3)	62 (60.8)	0.40 (0.20-0.70)	-	-	-
Nackers F et al. [38]	Case-control	Questionnaire/ Record/Scar	-	180 (64.5)	664 (67.2)	0.89 (0.67-1.17)*	-	Socioeconomic status and environmental exposure*	-
				176 (71.8)	592 (76.6)	0.79 (0.57-1.10)*	-		Subanalysis excluding subjects with no BCG scar/record.
Phillips RO et al. [28]	Case-control	Scar	BCG-Denmark, BCG-Japan, BCG-Russia and possibly others	226 (56.36)	549 (66.46)	0.65 (0.51-0.83)*	-	Age and country	Loss of significance of results upon stratification by age group, sex and country.
Pouillot R et al. [42]	Case-control	Record/Scar	-	96 (59)	114 (70)	0.61 (0.38-0.97)	-	-	Community-matched comparison.
				71 (60)	84 (71)	0.62 (0.36-1.1)	-		Familial-matched comparison.
Quek TYJ et al. [29]	Case-control	Questionnaire	-	16 (33)	174 (29)	1.22 (0.45-2.29)*	-	-	-
Raghunathan PL et al. [16]	Case-control	Scar	-	63 (54)	56 (48)	1.21 (0.65-2.26)*	-	-	-
Smith PG et al. [43]	RCT	Tuberculin	Glaxo Laboratories Ltd., Greenford, England	34 (1.92)	66 (2.39)	0.80 (0.53-1.22)*	-	Age and sex	-
Uganda Buruli Group [45]	RCT	Tuberculin	Glaxo Laboratories Ltd., Greenford, England	21 (3.46)	44 (7.05)	0.49 (0.29-0.84)*	-	Age and sex	-

*Calculated from the available data.
