# Supplementary material for: Individual and clinical variables associated with the risk of Buruli ulcer acquisition: A systematic review and meta-analysis
Source: PLoS Negl Trop Dis. 2020 Apr 8;14(4):e0008161. doi: 10.1371/journal.pntd.0008161 (PMC7170268; doi:10.1371/journal.pntd.0008161)
Supplement: S6 Table — (PDF) [file pntd.0008161.s008.pdf]

S6 Table. Human Immunodeficiency Virus (HIV) infection-related comparisons in case-control studies.

Study first author [reference]	HIV confirmation method	HIV^+ cases (%)	HIV^+ controls (%)	Crude OR (95% CI)	Adjusted OR (95% CI)	Confounders included in adjusted estimates	Observations
Johnson RC et al. [20]	Enzymatic immunoassay for HIV-1/2, confirmed with kits from different laboratories	6 (2.3)	2 (0.3)	7.3 (1.30–74.00)	8.9 (1.8–44)	Age, type of lesion	-
Raghunathan PL et al. [16]	Enzymatic immunoassay for HIV-1/2; western blot confirmation for HIV-1	6 (5)	1 (0.9)	6.27 (0.74-52.95)	-	-	-
Stienstra Y et al. [44]	Enzymatic immunoassay for HIV-1/2; western blot for HIV-1/2 and recombinant immunoblot assay for confirmation	5 (4.7)	1 (0.9)	5.20 (0.60-45.27)*	-	-	$p = 0.212$ (Fisher's exact test)

*Calculated from the available data.
