# Supplementary material for: Individual and clinical variables associated with the risk of Buruli ulcer acquisition: A systematic review and meta-analysis
Source: PLoS Negl Trop Dis. 2020 Apr 8;14(4):e0008161. doi: 10.1371/journal.pntd.0008161 (PMC7170268; doi:10.1371/journal.pntd.0008161)
Supplement: S7 Table — (PDF) [file pntd.0008161.s009.pdf]

S7 Table. Risk of bias among the cohort studies included in the systematic review.

Author [reference]	Year	1. Were the two groups similar and recruited from the same population?	2. Were the exposures measured similarly to assign people to both exposed and unexposed groups?	3. Was the exposure measured in a valid and reliable way?	4. Were confounding factors identified?	5. Were strategies to deal with confounding factors stated?	6. Were the groups/participants free of the outcome at the start of the study (or at the moment of exposure)?	7. Were the outcomes measured in a valid and reliable way?	8. Was the follow up time reported and sufficient to be long enough for outcomes to occur?	9. Was follow up complete, and if not, were the reasons to loss to follow up described and explored?	10. Were strategies to address incomplete follow up utilized?	11. Was appropriate statistical analysis used?	Overall appraisal
Bratschi MW et al. [31]	2013	Not applicable	Not applicable	Yes	No	No	Unclear	Yes	Yes	Not applicable	Not applicable	Yes	Include
Debacker M et al. [10]	2004	Not applicable	Not applicable	Yes	No	No	Unclear	Unclear	Yes	Not applicable	Not applicable	Yes	Include
Douine M et al. [4]	2017	Not applicable	Not applicable	Yes	No	No	Unclear	Unclear	Yes	Not applicable	Not applicable	Yes	Include
Johnson PDR et al. [34]	2007	Not applicable	Not applicable	Yes	No	No	Unclear	Yes	Yes	Not applicable	Not applicable	Yes	Include
Landier J et al. [36]	2014	Not applicable	Not applicable	Unclear	No	No	Unclear	Yes	Yes	Not applicable	Not applicable	Yes	Include
O'Brien DP et al. [41]	2017	Not applicable	Not applicable	Yes	No	No	Unclear	Yes	Yes	Not applicable	Not applicable	Yes	Include
Uganda Buruli Group [45]	1971	Not applicable	Not applicable	Yes	No	No	Unclear	Unclear	Yes	Not applicable	Not applicable	Yes	Include
