# Supplementary material for: Individual and clinical variables associated with the risk of Buruli ulcer acquisition: A systematic review and meta-analysis
Source: PLoS Negl Trop Dis. 2020 Apr 8;14(4):e0008161. doi: 10.1371/journal.pntd.0008161 (PMC7170268; doi:10.1371/journal.pntd.0008161)
Supplement: S8 Table — (PDF) [file pntd.0008161.s010.pdf]

S8 Table. Risk of bias among the case-control studies included in meta-analysis.

Author [reference]	Year	1. Were the groups comparable other than the presence of disease in cases or the absence of disease in controls?	2. Were cases and controls matched appropriately?	3. Were the same criteria used for identification of cases and controls?	4. Was exposure measured in a standard, valid and reliable way?	5. Was exposure measured in the same way for cases and controls?	6. Were confounding factors identified?	7. Were strategies to deal with confounding factors stated?	8. Were outcomes assessed in a standard, valid and reliable way for cases and controls?	9. Was the exposure period of interest long enough to be meaningful?	10. Was appropriate statistical analysis used?	Overall appraisal
Ahoua L et al. [18]	2009	Yes	Yes	Yes	No	Yes	Unclear	Yes	Unclear	No	Yes	Include
Aiga H et al. [32]	2004	No	No	Unclear	Unclear	Yes	Unclear	Yes	Unclear	No	Yes	Include
Bibert S et al. [13]	2017	Yes	Yes	Yes	Unclear	Unclear	Unclear	Yes	Yes	Unclear	Yes	Include
Capela C et al. [12]	2016	Yes	Yes	Unclear	Unclear	Unclear	Unclear	Yes	Yes	Yes	Yes	Include
Debacker M et al. [33]	2006	No	No	Unclear	Yes	Unclear	Unclear	Yes	Unclear	Yes	Yes	Include
Johnson RC et al. [20]	2008	Yes	Yes	Yes	Yes	Yes	Unclear	Yes	Yes	Yes	Yes	Include
Kenu E et al. [35]	2014	Yes	Yes	Yes	Unclear	Yes	Unclear	Yes	Yes	Yes	Yes	Include
Maman I et al. [30]	2018	Yes	No	Yes	Unclear	Yes	Unclear	Yes	Yes	Yes	Yes	Include
Marston BJ et al. [11]	1995	Yes	No	Yes	Unclear	Yes	Unclear	Yes	Unclear	Yes	Yes	Include
N'krumah RTAS et al. [37]	2016	Unclear	Yes	Yes	Unclear	Yes	Unclear	Yes	Yes	No	Yes	Include
Nackers F et al. [38]	2006	Yes	Yes	Yes	Unclear	Yes	Unclear	Yes	Unclear	Yes	Yes	Include
Nackers F et al. [39]	2007	Yes	Yes	Yes	Yes	Yes	Unclear	Yes	Yes	Yes	Yes	Include
Nackers F et al. [40]	2007	Yes	Yes	Yes	Yes	Yes	Unclear	Yes	Yes	Yes	Yes	Include
Phillips RO et al. [28]	2015	Yes	No	Yes	Unclear	Yes	Unclear	Yes	Yes	Yes	Yes	Include
Pouillot R et al. [42]	2007	Yes	No	Yes	Unclear	Yes	Unclear	Yes	Unclear	No	Yes	Include
Quek TYJ et al. [29]	2007	Unclear	No	Yes	No	Yes	Unclear	Yes	Unclear	Unclear	Yes	Include
Raghunathan PL et al. [16]	2005	Yes	Yes	Yes	Unclear	Yes	Unclear	Yes	Yes	No	Yes	Include
Sopoh GE et al. [15]	2010	Yes	Yes	Yes	Yes	Yes	Unclear	Yes	Yes	Yes	Yes	Include
Stienstra Y et al. [44]	2004	Yes	Yes	Yes	Yes	Yes	Unclear	No	Yes	No	Yes	Include
Stienstra Y et al. [14]	2006	Yes	No	Yes	Yes	Yes	Unclear	Yes	Unclear	Yes	Yes	Include
