# Supplementary material for: Individual and clinical variables associated with the risk of Buruli ulcer acquisition: A systematic review and meta-analysis
Source: PLoS Negl Trop Dis. 2020 Apr 8;14(4):e0008161. doi: 10.1371/journal.pntd.0008161 (PMC7170268; doi:10.1371/journal.pntd.0008161)
Supplement: S9 Table — (PDF) [file pntd.0008161.s011.pdf]

S9 Table. Risk of bias among the RCTs included in meta-analysis.

Author [reference]	Year	1. Was true randomization used for assignment of participants to treatment groups?	2. Was allocation to treatment groups concealed?	3. Were treatment groups similar at the baseline?	4. Were participants blind to treatment assignment?	5. Were those delivering treatment blind to treatment assignment?	6. Were outcomes assessors blind to treatment assignment?	7. Were treatment groups treated identically other than the intervention of interest?	8. Was follow up complete and if not, were differences between groups in terms of their follow up adequately described and analyzed?	9. Were participants analyzed in the groups to which they were randomized?	10. Were outcomes measured in the same way for treatment groups?	11. Were outcomes measured in a reliable way?	12. Was appropriate statistical analysis used?	13. Was the trial design appropriate, and any deviations from the standard RCT design (individual randomization, parallel groups) accounted for in the conduct and analysis of the trial?	Overall appraisal
Smith PG et al. [43]	1976	Yes	No	Yes	No	No	No	Yes	Unclear	Yes	Yes	Yes	Yes	No	Include
Uganda Buruli Group [45]	1969	Yes	No	Yes	No	No	No	Yes	Unclear	Yes	Yes	Yes	Yes	No	Include
