# Supplementary material for: Individual and clinical variables associated with the risk of Buruli ulcer acquisition: A systematic review and meta-analysis
Source: PLoS Negl Trop Dis. 2020 Apr 8;14(4):e0008161. doi: 10.1371/journal.pntd.0008161 (PMC7170268; doi:10.1371/journal.pntd.0008161)
Supplement: S10 Table — Overall male:female ratio = 1.06. (PDF) [file pntd.0008161.s012.pdf]

S10 Table. Demographic information from relevant observational and case series studies not eligible for systematic review. Overall male:female ratio = 1.06.

Study first author [reference]	Study year	Study period	Country (Continent)	BU cases (n)	Sex (n)		Sex ratio (M:F)	Median age (years)	Age group (years)	n by age group		Sex ratio (M:F)
					Female	Male				Female	Male	
Abass KM et al. [1]	2015	2009 - 2013	Ghana (Africa)	451	239	212	0.88	-	<16	262		-
									≥16	189		
Ackumey M et al. [2]	2011	Jun 2005 - Jun 2008<br>Nov 2008 - Jul 2009	Ghana (Africa)	297	153	144	0.94	-	<15	146		-
									15-49	128		
									>49	23		
Amofah G et al. [3]	2002	Jun - Jul 1999	Ghana (Africa)	5619	2742	2854	1.04	25	-	-		-
Ampah KA et al. [4]	2016	Feb 2013 - Dec 2014	Ghana (Africa)	477	266	211	0.79	-	<15	230		-
									≥15	250		
Ayelo GA et al. [5]	2018	Jan 2006 - Dec 2016	Benin (Africa)	82	37	45	1.22	20	-	-		-
Bafende AE et al. [6]	2004	1989 - 1999	DR Congo (Africa)	45	20	25	1.25	-	-	-		-
		2000 - 2001		62	-	-	-	-	≤15	31		-
									16-20	27		
									>60	4		
		Jan - Oct 2002		14	-	-	-	-	<15	9		-
									≥15	5		
Barker DJ et al. [7]	1973	Jan 1969 - Jul 1972	Uganda (Africa)	45	30	15	0.50	-	0-4	9	4	0.44
									5-9	3	12	4.00
									10-14	0	3	-
									≥15	3	11	3.67
Bayonne Manou LS et al. [8]	2013	Apr 2005 - Dec 2011	Gabon (Africa)	301	129	172	1.33	-	<15	103		-
									15-49	127		
									>49	71		
Boyd SC et al. [9]	2012	Jan 1998 - Sep 2011	Australia (Oceania)	180	91	89	0.98	61	<15	15		-
									15-60	72		
									>60	93		
Bretzel G et al. [10]	2011	Sep 2007 - Aug 2010	Togo (Africa)	202<br>109*	88<br>52*	114<br>57*	1.30<br>1.10*	17<br>12*	0-4	7*		-
									5-9	37		
									10-14	28*		
									15-19	9*		

									20-24	3*		
									25-29	3*		
									30-34	3*		
									35-39	3*		
									40-44	7*		
									45-49	1*		
									50-54	3*		
									55-59	3*		
									>59	4*		
Capela C et al. [11]	2015	Jan 2005 - Dec 2013	Benin (Africa)	476	231	245	1.06	12	≤15	128	193	1.51
									>15	103	52	0.50
Hospers IC et al. [12]	2005	1996 - 2002	Ghana (Africa)	748	408	340	0.83	12	<16	255	240	0.94
									≥16	152	100	0.658
Iabichella ML et al. [13]	2015	2005 - 2009	Benin (Africa)	171	104	67	0.64	15 or 18, depending on the BU center	-	-	-	-
Igo JD et al. [14]	1998	1979 - 1983	Papua New Guinea (Africa)	46	24	22	0.90	-	≤10	32		-
									>10	14		-
James K et al. [15]	2003	Jan 2000 - Dec 2001	Togo (Africa)	180	78	102	1.3	-	0-10	5	7	1.40
									11-20	19	22	1.15
									21-30	16	32	2.00
									31-40	15	21	1.40
									41-50	8	5	0.62
									51-60	8	9	1.12
									>60	7	6	0.85
Johnson RC et al. [16]	2005	2004	Benin (Africa)	752	-	-	-	-	<15	300		-
									15-49	363		
									>49	89		
Johnson RC et al. [17]	2008	2003 - 2006	Benin (Africa)	3793	-	-	0.98	14	<15	1995		-
									≥15	1798		
Kanga JM et al. [18]	2001	1978 - 1997	Ivory Coast (Africa)	10382	-	-	1.10	-	<15	2929	2939	1.00
									≥15	2781	1733	0.62
Lavender CJ et al. [19]	2011	Jan 2005 - Dec 2009	Australia (Oceania)	183	77	106	1.38	61	-	-		-

Loftus MJ et al. [20]	2018	Jan 2011 - Dec 2016	Australia (Oceania)	600	342	258	0.75	54	<15	72		-
									15-60	285		-
									≥60	243		-
Marion E et al. [21]	2014	2007 - 2012	DR Congo (Africa)	108	60	48	0.80	-	<15	60		-
									≥15	48		
Marion E et al. [22]	2015	2004 - 2013	Benin (Africa)	127	66	61	0.92	-	≤15	68		-
									>15	59		
Mavinga Phanzu D et al. [23]	2013	Jul - Aug 2008	DR Congo (Africa)	775<br>72*	465<br>44*	310<br>28*	0.67<br>0.64*	27	<15	27*		-
									16-49	35*		
									>49	10*		
Meka AO et al. [24]	2016	May 2014 - Sep 2015	Nigeria (Africa)	145	85	60	0.71	20	≤15	34	23	0.68
									>15	51	37	0.73
Mensah-Quainoo E et al. [25]	2008	Jul 2001 - Jul 2004	Ghana (Africa)	99	57	42	0.74	11	0-4	4	6	1.50
									5-9	17	10	0.59
									10-14	14	11	0.79
									15-19	6	7	1.17
									20-24	3	3	1.00
									25-29	3	1	0.33
									30-34	0	0	-
									35-39	2	0	-
									40-44	2	1	0.50
									45-49	2	1	0.50
									50-54	0	1	-
									55-59	2	0	-
									>60	2	1	0.50
N'Krumah RTAS et al. [26]	2017	2005 - 2010	Ivory Coast (Africa)	1145	562	583	1.04	-	<15	275	332	1.21
									15-49	244	206	0.84
									≥50	43	45	1.05
Ngoa UA et al. [27]	2012	2001 - 2010	Gabon (Africa)	77	-	-	0.83	-	<15	40		-
									≥15	30		
Noeske J et al. [28]	2004	Aug 2001	Cameroon (Africa)	202	87	115	1.32	14.5	<15	101		-

									≥15	101		
O'Brien DP et al. [29]	2015	Jan 1998 - Dec 2014	Australia (Oceania)	327	162	165	1.02	58	<65	87	109	1.25
									≥65	75	56	0.75
									>15	3	7	2.33
Phanzu DM et al. [30]	2006	May 2002 - Aug 2004	DR Congo (Africa)	36	9	27	3.00	17	≤15	16		-
									16-45	13		
									>45	7		
Phanzu DM et al. [31]	2011	Jan 2002 - Dec 2007	DR Congo (Africa)	254	113	141	1.25	20	≤15	99		-
									16-45	104		
									>45	51		
Porten K et al. [32]	2009	Mar 2007	Cameroon (Africa)	105	46	59	1.28	15.5	-	-		-
Quek TYJ et al. [33]	2007	Jan 1998 - Aug 2016	Oceania (Austrailia)	85	40	45	1.13	-	<60	32		-
									≥60	53		
Saka B et al. [34]	2013	Jun 2007 - Dec 2010	Togo (Africa)	119	52	67	1.29	14	<15	67		-
									≥15	52		
Smith JH [35]	1970	1961 - 1968	DR Congo (Africa)	97	46	48	1.04	-	<5	6	7	1.17
									5-9	15	14	0.93
									10-14	8	11	1.38
									≥15	17	16	0.94
Sopoh GE et al. [36]	2007	Jan 2003 - Dec 2005	Benin (Africa)	2598	1307	1291	0.99	-	<15	1325		-
									≥15	1273		
Steffen CM et al. [37]	2018	Jan 2009 - Dec 2015	Australia (Oceania)	95	41	54	1.32	45	-	-		-
Suykerbuyk P et al. [38]	2009	Oct 2007	DR Congo (Africa)	75	35	40	1.14	23	5-9	2	4	2
									10-14	6	9	1.5
									15-29	4	6	1.5
									20-24	5	2	0.4
									25-29	5	2	0.4
									30-34	4	4	1
									35-39	2	6	3
									40-44	1	1	1
									45-49	1	1	1

									50-54	1	0	-
									55-59	0	3	-
									60-64	0	1	-
									>65	4	1	0.25
Tai AYC et al. [39]	2018	Jan 1998 - May 2017	Australia (Oceania)	426	196	230	1.17	58	≤15	40		-
									16-64	217		
									≥65	169		
Toutous Trellu L et al. [40]	2016	Oct 2011 - Dec 2013	Cameroon (Africa)	87	41	46	1.12	20	<15	35		-
									15-44	34		
									≥45	18		
Tuffour J et al. [41]	2015	Oct 2009 - Mar 2013	Ghana (Africa)	134	67	67	1	-	≤15	36		-
									>15	98		
Ukwaja KN et al. [42]	2016	May 2012 - Apr 2013	Nigeria (Africa)	36	24	12	0.50	17	≤15	15		-
									>15	21		
Vincent QB et al. [43]	2014	Jan 2005 - Dec 2011	Benin (Africa)	1227	640	587	0.91	12	≤15	322	427	1.33
									>15	306	158	0.51
Wu J et al. [44]	2015	2007 - 2010	Ghana (Africa)	339	167	172	1.03	-	0-4	11	18	1.64
									5-9	16	18	1.13
									10-14	17	15	0.88
									15-19	7	17	2.43
									20-24	15	8	0.53
									25-29	13	8	0.62
									30-34	8	13	1.63
									35-39	9	9	1.00
									40-44	10	7	0.70
									45-49	10	8	0.80
									50-54	7	11	1.57
									55-59	2	8	4.00
									60-64	11	6	0.55
									65-69	6	9	1.50
									70-74	14	6	0.43
									75-79	6	6	1.00

									80-84	2	1	0.50
									85+	3	4	1.33
Yeboah-Manu D et al. [45]	2018	2008 - 2016	Ghana (Africa)	2203<br>1020*	1086<br>518*	1117<br>502*	1.03<br>0.97*	22<br>20*	0-15	880<br>406*		-
									16-30	398<br>188*		
									31-45	342<br>158*		
									46-60	246<br>115*		
									≥61	239<br>108*		
Yerramilli A et al. [46]	2017	1998 - 2015	Australia (Oceania)	585	266	319	1.20	55	≤15	75		-
									15-64	301		
									≥65	209		

*Subset of BU patients with laboratory-confirmed BU disease.

S10 Table References.

1. Abass KM, van der Werf TS, Phillips RO, Sarfo FS, Abotsi J, Mireku SO, et al. Buruli ulcer control in a highly endemic district in Ghana: role of community-based surveillance volunteers. Am J Trop Med Hyg. 2015;92: 115–117. doi:10.4269/ajtmh.14-0405
2. Ackumey MM, Kwakye-Maclean C, Ampadu EO, de Savigny D, Weiss MG. Health services for Buruli ulcer control: lessons from a field study in Ghana. PLoS Negl Trop Dis. 2011;5: e1187. doi:10.1371/journal.pntd.0001187
3. Amofah G, Bonsu F, Tetteh C, Okrah J, Asamoa K, Asiedu K, et al. Buruli ulcer in Ghana: results of a national case search. Emerg Infect Dis. 2002;8: 167–170. doi:10.3201/eid0802.010119
4. Ampah KA, Asare P, Binnah DD-G, Maccaulley S, Opare W, Röltgen K, et al. Burden and Historical Trend of Buruli Ulcer Prevalence in Selected Communities along the Offin River of Ghana. PLoS Negl Trop Dis. 2016;10: e0004603. doi:10.1371/journal.pntd.0004603
5. Ayelo GA, Anagonou E, Wadagni AC, Barogui YT, Dossou AD, Houezo JG, et al. Report of a series of 82 cases of Buruli ulcer from Nigeria treated in Benin, from 2006 to 2016. PLoS Negl Trop Dis. 2018;12: e0006358. doi:10.1371/journal.pntd.0006358
6. Bafende AE, Phanzu MD, Imposo BB. Buruli ulcer in the Democratic Republic of Congo: epidemiology, presentation and outcome. Trop Doct. 2004;34: 82–84. doi:10.1177/004947550403400207
7. Barker DJ. Epidemiology of Mycobacterium ulcerans infection. Trans R Soc Trop Med Hyg. 1973;67: 43–50.
8. Bayonne Manou LS, Portaels F, Eddyani M, Book AU, Vandelannoote K, de Jong BC. [Mycobacterium ulcerans disease (Buruli ulcer) in Gabon: 2005-2011]. Med Sante Trop. 2013;23: 450–457. doi:10.1684/mst.2013.0259
9. Boyd SC, Athan E, Friedman ND, Hughes A, Walton A, Callan P, et al. Epidemiology, clinical features and diagnosis of Mycobacterium ulcerans in an Australian population. Med J Aust. 2012;196: 341–344.
10. Bretzel G, Huber KL, Kobara B, Beissner M, Piten E, Herbinger K-H, et al. Laboratory confirmation of Buruli ulcer disease in Togo, 2007-2010. PLoS Negl Trop Dis. 2011;5: e1228. doi:10.1371/journal.pntd.0001228
11. Capela C, Dossou AD, Silva-Gomes R, Sopoh GE, Makoutode M, Menino JF, et al. Genetic Variation in Autophagy-Related Genes Influences the Risk and Phenotype of Buruli Ulcer. PLoS Negl Trop Dis. 2016;10: e0004671. doi:10.1371/journal.pntd.0004671
12. Hospers IC, Wiersma IC, Dijkstra PU, Stienstra Y, Etuaful S, Ampadu EO, et al. Distribution of Buruli ulcer lesions over body surface area in a large case series in Ghana: uncovering clues for mode of transmission. Trans R Soc Trop Med Hyg. 2005;99: 196–201. doi:10.1016/j.trstmh.2004.05.004
13. Iabichella ML, Salmon O, Bertolotti A, Izzo A, Fusari V, Lugli M. Buruli ulcer: Management in hospital or at public health centers in “brousse.” Angeiologie. 2015;67: 29–41.
14. Igo JD, Murthy DP. Mycobacterium ulcerans infections in Papua New Guinea: correlation of clinical, histological, and microbiologic features. Am J Trop Med Hyg. 1988;38: 391–392.
15. James K, Attipou KK, James YE, Blakime M, Tignokpa N, Abete B. L’ulcère de Buruli au Togo : à propos d’une enquête hospitalière. Cah Détudes Rech Francoph Santé. 2003;13: 43–47.
16. Johnson RC, Sopoh GE, Boko M, Zinsou C, Gbovi J, Makoutode M, et al. [Distribution of Mycobacterium ulcerans (Buruli ulcer) in the district of Lalo in Benin]. Trop Med Int Health TM IH. 2005;10: 863–871. doi:10.1111/j.1365-3156.2005.01465.x
17. Johnson RC, Sopoh GE, Barogui Y, Dossou A, Fourn L, Zohoun T. [Surveillance system for Buruli ulcer in Benin: results after four years]. Sante Montrouge Fr. 2008;18: 9–13. doi:10.1684/san.2008.0098
18. Kanga JM, Kacou ED. [Epidemiologicl aspects of Buruli ulcer in Côte d’Ivoire: results of a national survey]. Bull Soc Pathol Exot 1990. 2001;94: 46–51.
19. Lavender CJ, Fyfe JAM, Azuolas J, Brown K, Evans RN, Ray LR, et al. Risk of Buruli ulcer and detection of Mycobacterium ulcerans in mosquitoes in southeastern Australia. PLoS Negl Trop Dis. 2011;5: e1305. doi:10.1371/journal.pntd.0001305
20. Loftus MJ, Tay EL, Globan M, Lavender CJ, Crouch SR, Johnson PDR, et al. Epidemiology of Buruli Ulcer Infections, Victoria, Australia, 2011-2016. Emerg Infect Dis. 2018;24: 1988–1997. doi:10.3201/eid2411.171593
21. Marion E, Obvala D, Babonneau J, Kempf M, Asiedu KB, Marsollier L. Buruli ulcer disease in Republic of the Congo. Emerg Infect Dis. 2014;20: 1070–1072. doi:10.3201/eid2006.131498
22. Marion E, Carolan K, Adeye A, Kempf M, Chauty A, Marsollier L. Buruli ulcer in South Western Nigeria: a retrospective cohort study of patients treated in Benin. PLoS Negl Trop Dis. 2015;9: e3443. doi:10.1371/journal.pntd.0003443

23. Mavinga Phanzu D, Suykerbuyk P, Saunderson P, Ngwala Lukanu P, Masamba Minuku J-B, Imposo DBB, et al. Burden of Mycobacterium ulcerans disease (Buruli ulcer) and the underreporting ratio in the territory of Songololo, Democratic Republic of Congo. PLoS Negl Trop Dis. 2013;7: e2563. doi:10.1371/journal.pntd.0002563
24. Meka AO, Chukwu JN, Nwafor CC, Oshi DC, Madichie NO, Ekeke N, et al. Diagnosis delay and duration of hospitalisation of patients with Buruli ulcer in Nigeria. Trans R Soc Trop Med Hyg. 2016;110: 502–509. doi:10.1093/trstmh/trw065
25. Mensah-Quainoo E, Yeboah-Manu D, Asebi C, Patafuor F, Ofori-Adjei D, Junghanss T, et al. Diagnosis of Mycobacterium ulcerans infection (Buruli ulcer) at a treatment centre in Ghana: a retrospective analysis of laboratory results of clinically diagnosed cases. Trop Med Int Health TM IH. 2008;13: 191–198. doi:10.1111/j.1365-3156.2007.01990.x
26. N'krumah RTAS, Koné B, Cissé G, Tanner M, Utzinger J, Pluschke G, et al. Characteristics and epidemiological profile of Buruli ulcer in the district of Tiassalé, south Côte d'Ivoire. Acta Trop. 2017;175: 138–144. doi:10.1016/j.actatropica.2016.12.023
27. Ngoa UA, Adzoda GK, Louis BM, Adegnika AA, Lell B. Buruli ulcer in Gabon, 2001-2010. Emerg Infect Dis. 2012;18: 1206–1207. doi:10.3201/eid1807.110613
28. Noeske J, Kuaban C, Rondini S, Sorlin P, Ciaffi L, Mbuagbaw J, et al. Buruli ulcer disease in Cameroon rediscovered. Am J Trop Med Hyg. 2004;70: 520–526.
29. O'Brien DP, Friedman ND, Cowan R, Pollard J, McDonald A, Callan P, et al. Mycobacterium ulcerans in the Elderly: More Severe Disease and Suboptimal Outcomes. PLoS Negl Trop Dis. 2015;9: e0004253. doi:10.1371/journal.pntd.0004253
30. Phanzu DM, Bafende EA, Dunda BK, Imposo DB, Kibadi AK, Nsiangana SZ, et al. Mycobacterium ulcerans disease (Buruli ulcer) in a rural hospital in Bas-Congo, Democratic Republic of Congo, 2002-2004. Am J Trop Med Hyg. 2006;75: 311–314.
31. Phanzu DM, Suykerbuyk P, Imposo DBB, Lukanu PN, Minuku J-BM, Lehman LF, et al. Effect of a control project on clinical profiles and outcomes in buruli ulcer: a before/after study in Bas-Congo, Democratic Republic of Congo. PLoS Negl Trop Dis. 2011;5: e1402. doi:10.1371/journal.pntd.0001402
32. Porten K, Sailor K, Comte E, Njikap A, Sobry A, Sihom F, et al. Prevalence of Buruli ulcer in Akonolinga health district, Cameroon: results of a cross sectional survey. PLoS Negl Trop Dis. 2009;3: e466. doi:10.1371/journal.pntd.0000466
33. Quek TYJ, Henry MJ, Pasco JA, O'Brien DP, Johnson PDR, Hughes A, et al. Mycobacterium ulcerans infection: factors influencing diagnostic delay. Med J Aust. 2007;187: 561–563.
34. Saka B, Landoh DE, Kobara B, Djadou KE, Yaya I, Yékplé KB, et al. [Profile of Buruli ulcer treated at the National Reference Centre of Togo: a study of 119 cases]. Bull Soc Pathol Exot 1990. 2013;106: 32–36. doi:10.1007/s13149-012-0241-1
35. Smith JH. Epidemiologic observations on cases of Buruli ulcer seen in a hospital in the Lower Congo. Am J Trop Med Hyg. 1970;19: 657–663.
36. Sopoh GE, Johnson RC, Chauty A, Dossou AD, Aguiar J, Salmon O, et al. Buruli ulcer surveillance, Benin, 2003-2005. Emerg Infect Dis. 2007;13: 1374–1376. doi:10.3201/eid1309.061338
37. Steffen CM, Freeborn H. Mycobacterium ulcerans in the Daintree 2009-2015 and the mini-epidemic of 2011. ANZ J Surg. 2018;88: E289–E293. doi:10.1111/ans.13817
38. Suykerbuyk P, Wambacq J, Phanzu DM, Haruna H, Nakazawa Y, Ooms K, et al. Persistence of Mycobacterium ulcerans disease (Buruli Ulcer) in the historical focus of Kasongo Territory, the Democratic Republic of Congo. Am J Trop Med Hyg. 2009;81: 888–894. doi:10.4269/ajtmh.2009.09-0049
39. Tai AYC, Athan E, Friedman ND, Hughes A, Walton A, O'Brien DP. Increased Severity and Spread of Mycobacterium ulcerans, Southeastern Australia. Emerg Infect Dis. 2018;24. doi:10.3201/eid2401.171070
40. Toutous Trellu L, Nkemenang P, Comte E, Ehounou G, Atangana P, Mboua DJ, et al. Differential Diagnosis of Skin Ulcers in a Mycobacterium ulcerans Endemic Area: Data from a Prospective Study in Cameroon. PLoS Negl Trop Dis. 2016;10: e0004385. doi:10.1371/journal.pntd.0004385
41. Tuffour J, Owusu-Mireku E, Ruf M-T, Aboagye S, Kpeli G, Akuoku V, et al. Challenges Associated with Management of Buruli Ulcer/Human Immunodeficiency Virus Coinfection in a Treatment Center in Ghana: A Case Series Study. Am J Trop Med Hyg. 2015;93: 216–223. doi:10.4269/ajtmh.14-0571
42. Ukwaja KN, Meka AO, Chukwuka A, Asiedu KB, Huber KL, Eddyani M, et al. Buruli ulcer in Nigeria: results of a pilot case study in three rural districts. Infect Dis Poverty. 2016;5: 39. doi:10.1186/s40249-016-0119-8

43. Vincent QB, Ardant M-F, Adeye A, Goundote A, Saint-André J-P, Cottin J, et al. Clinical epidemiology of laboratory-confirmed Buruli ulcer in Benin: a cohort study. Lancet Glob Health. 2014;2: e422-430. doi:10.1016/S2214-109X(14)70223-2
44. Wu J, Tschakert P, Klutse E, Ferring D, Ricciardi V, Hausermann H, et al. Buruli Ulcer Disease and Its Association with Land Cover in Southwestern Ghana. PLoS Negl Trop Dis. 2015;9: e0003840. doi:10.1371/journal.pntd.0003840
45. Yeboah-Manu D, Aboagye SY, Asare P, Asante-Poku A, Ampah K, Danso E, et al. Laboratory confirmation of Buruli ulcer cases in Ghana, 2008-2016. PLoS Negl Trop Dis. 2018;12: e0006560. doi:10.1371/journal.pntd.0006560
46. Yerramilli A, Tay EL, Stewardson AJ, Kelley PG, Bishop E, Jenkin GA, et al. The location of Australian Buruli ulcer lesions-Implications for unravelling disease transmission. PLoS Negl Trop Dis. 2017;11: e0005800. doi:10.1371/journal.pntd.0005800
