# Supplementary figures and images for: Individual and clinical variables associated with the risk of Buruli ulcer acquisition: A systematic review and meta-analysis
Source: PLoS Negl Trop Dis. 2020 Apr 8;14(4):e0008161. doi: 10.1371/journal.pntd.0008161 (PMC7170268; doi:10.1371/journal.pntd.0008161)

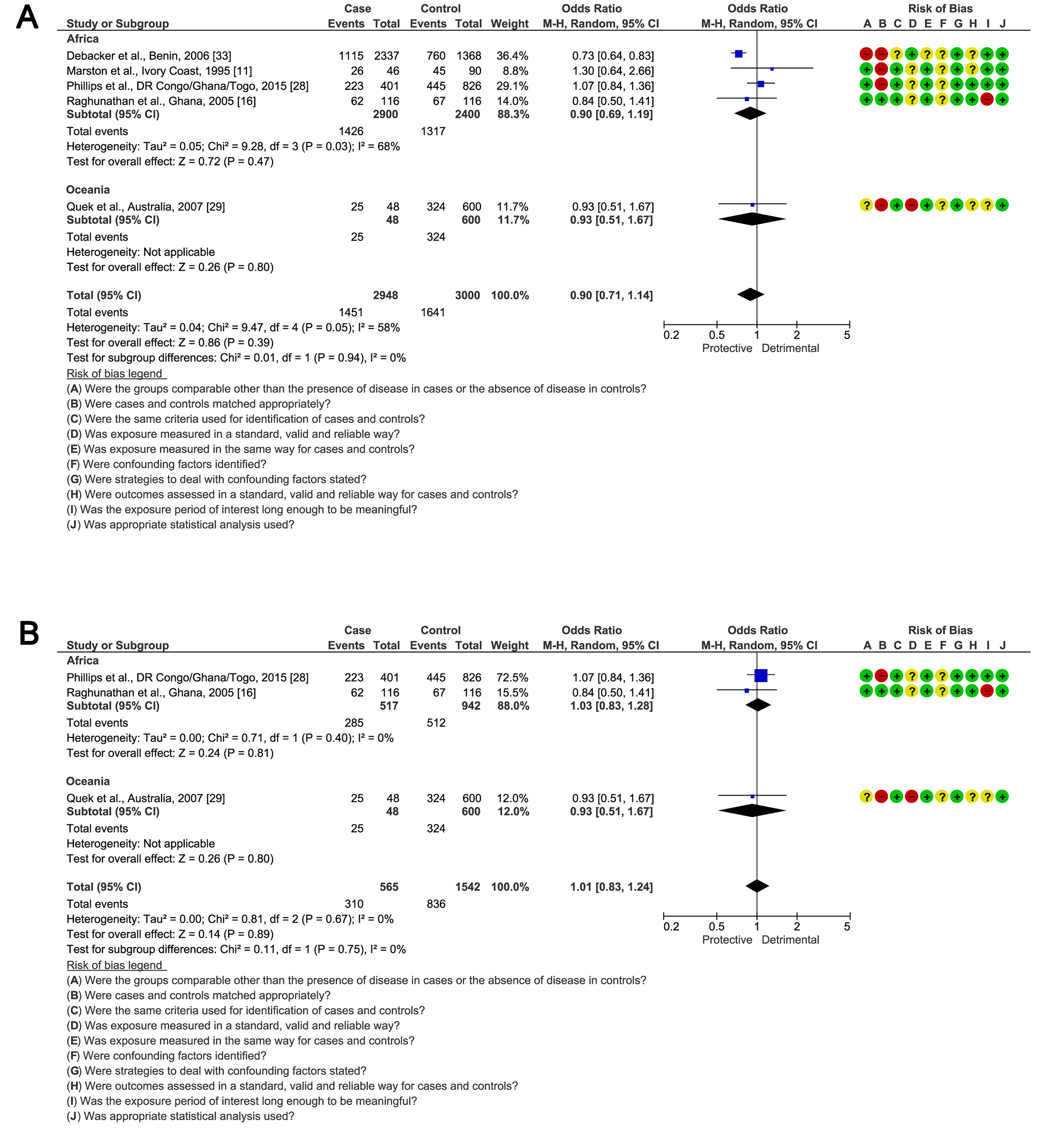

Supplement: S1 Fig — The number of events indicates female individuals. (A) All BU cases included. (B) BU laboratory-confirmed cases only. Bias: + low risk;—high risk; ? unclear risk. (TIF) [file pntd.0008161.s013.tif]

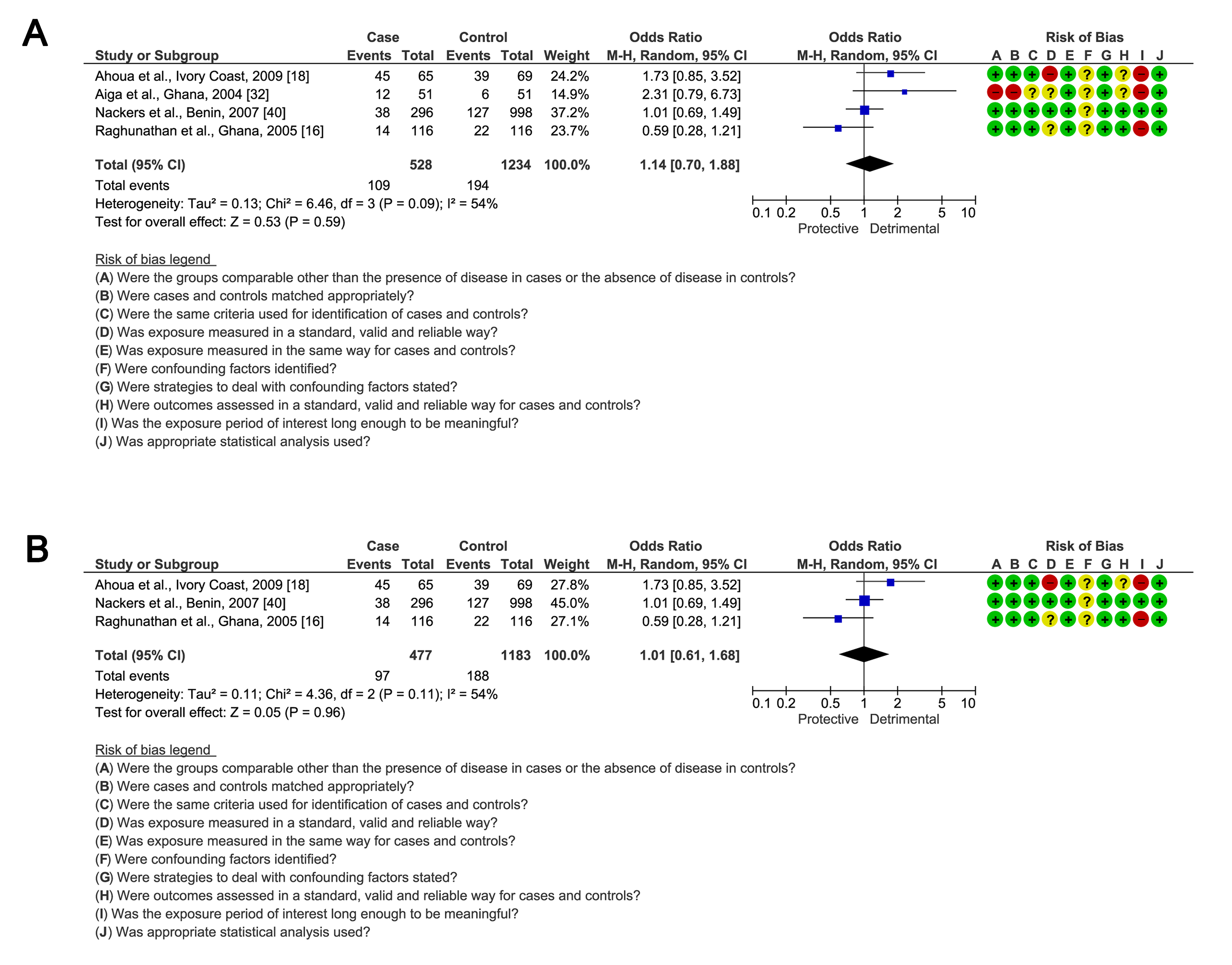

Supplement: S2 Fig — The number of events indicates patients with BU history in family members. (A) All BU cases included. (B) BU laboratory-confirmed cases only. Bias: + low risk;—high risk; ? unclear risk. (TIF) [file pntd.0008161.s014.tif]

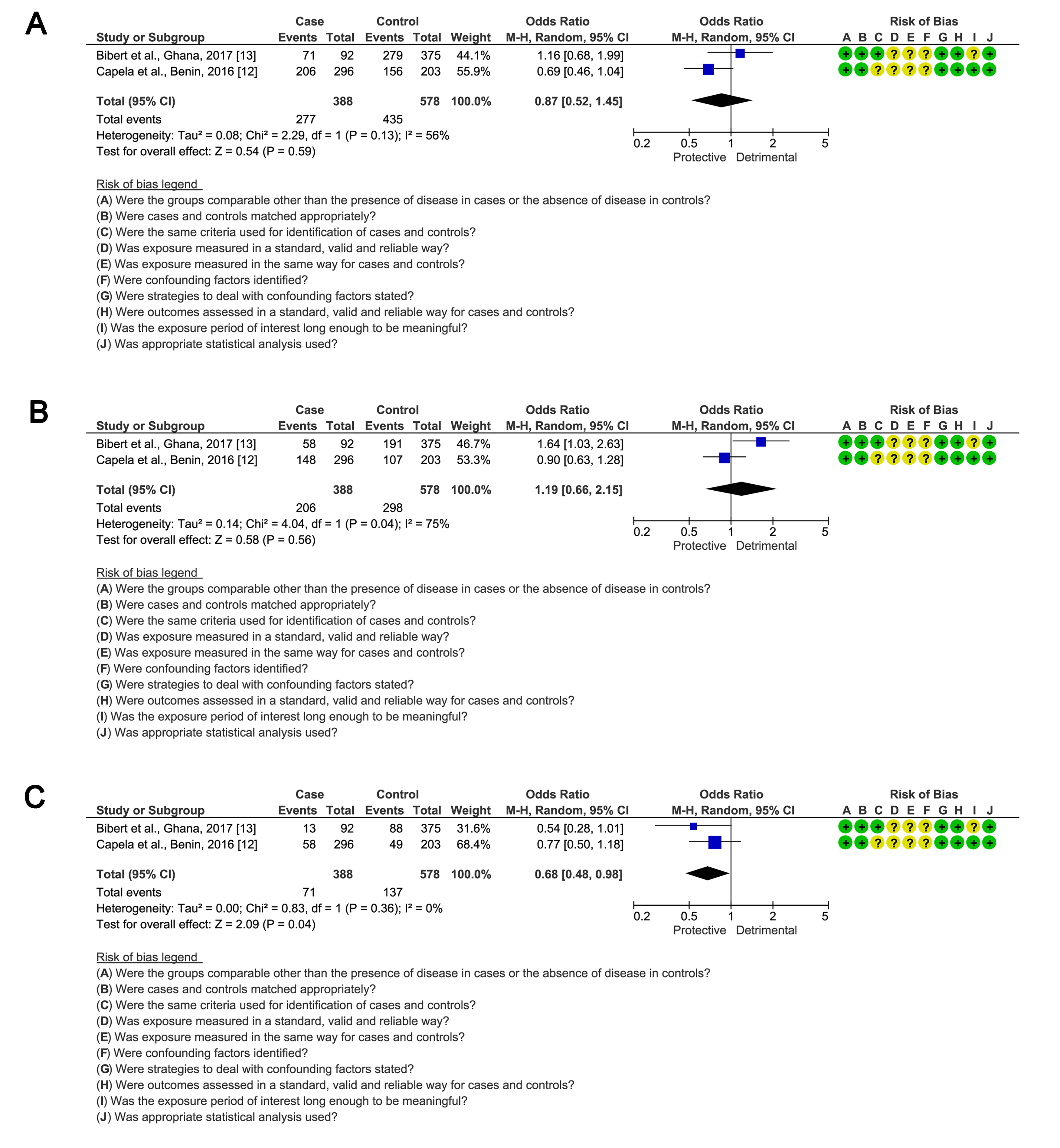

Supplement: S3 Fig — (A) Dominant. (B) Overdominant. (C) Recessive. (TIF) [file pntd.0008161.s015.tif]

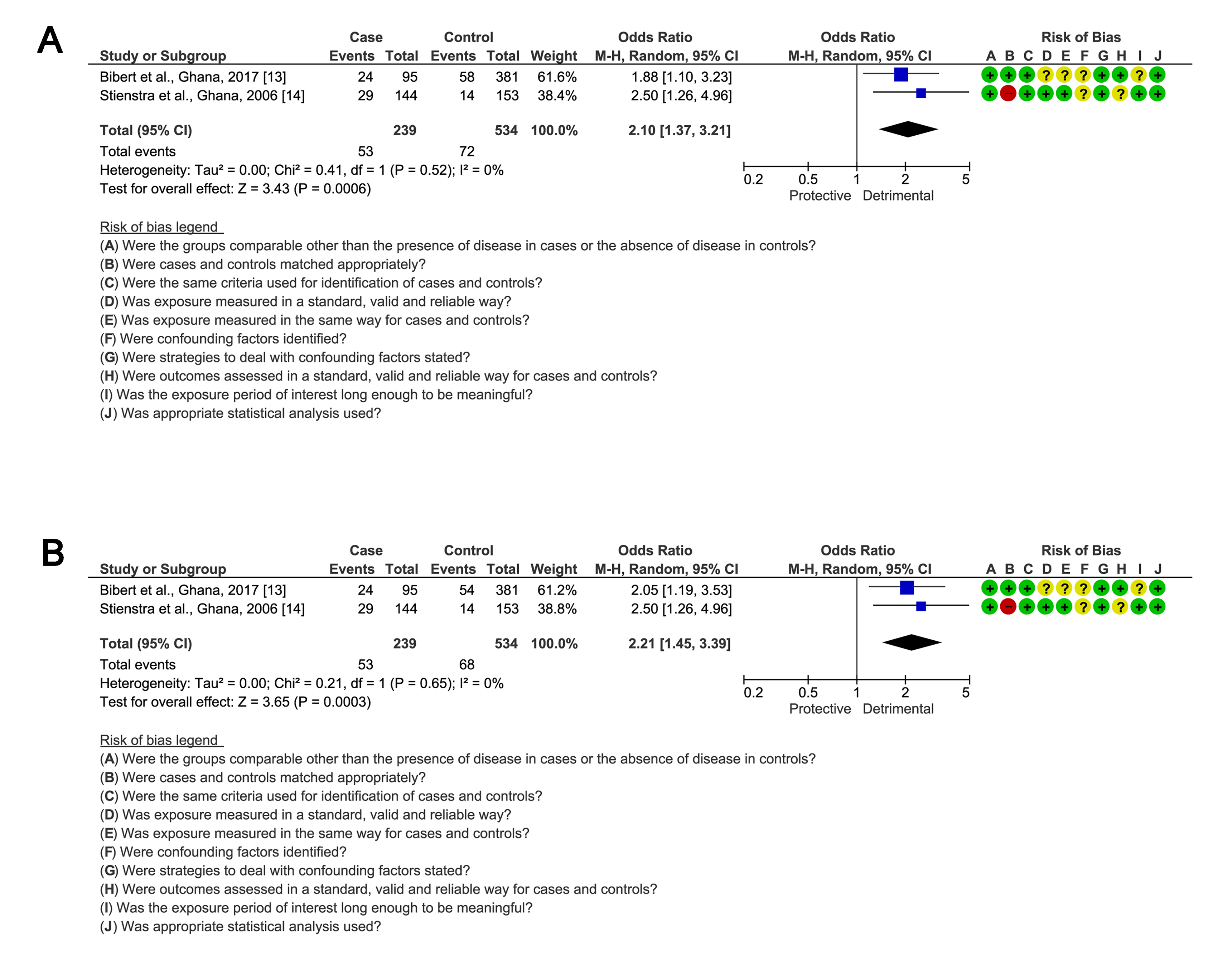

Supplement: S4 Fig — (A) Dominant. (B) Overdominant. (TIF) [file pntd.0008161.s016.tif]

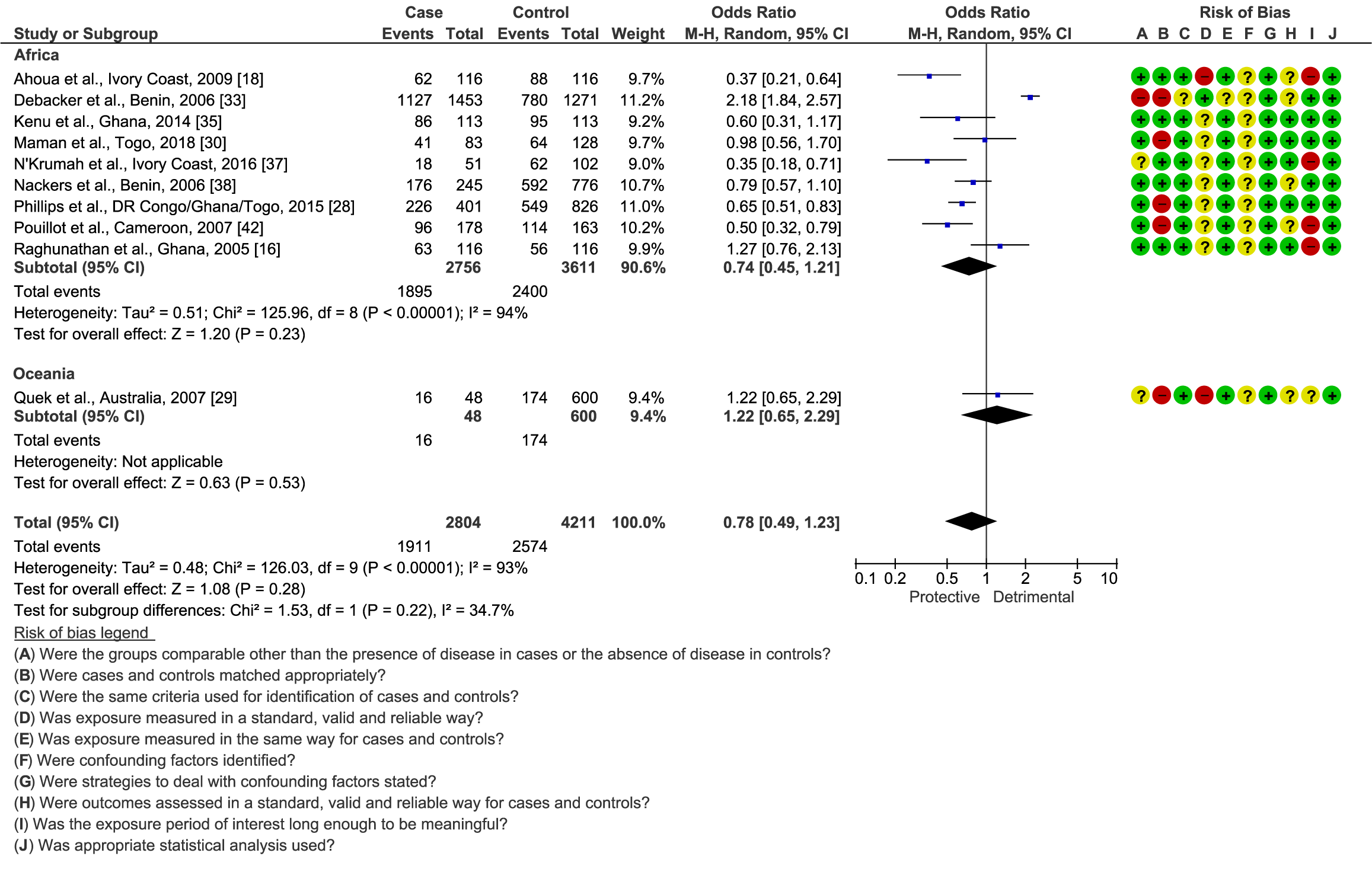

Supplement: S5 Fig — The number of events indicates BCG-vaccinated individuals. Bias: + low risk;—high risk; ? unclear risk. (TIF) [file pntd.0008161.s017.tif]

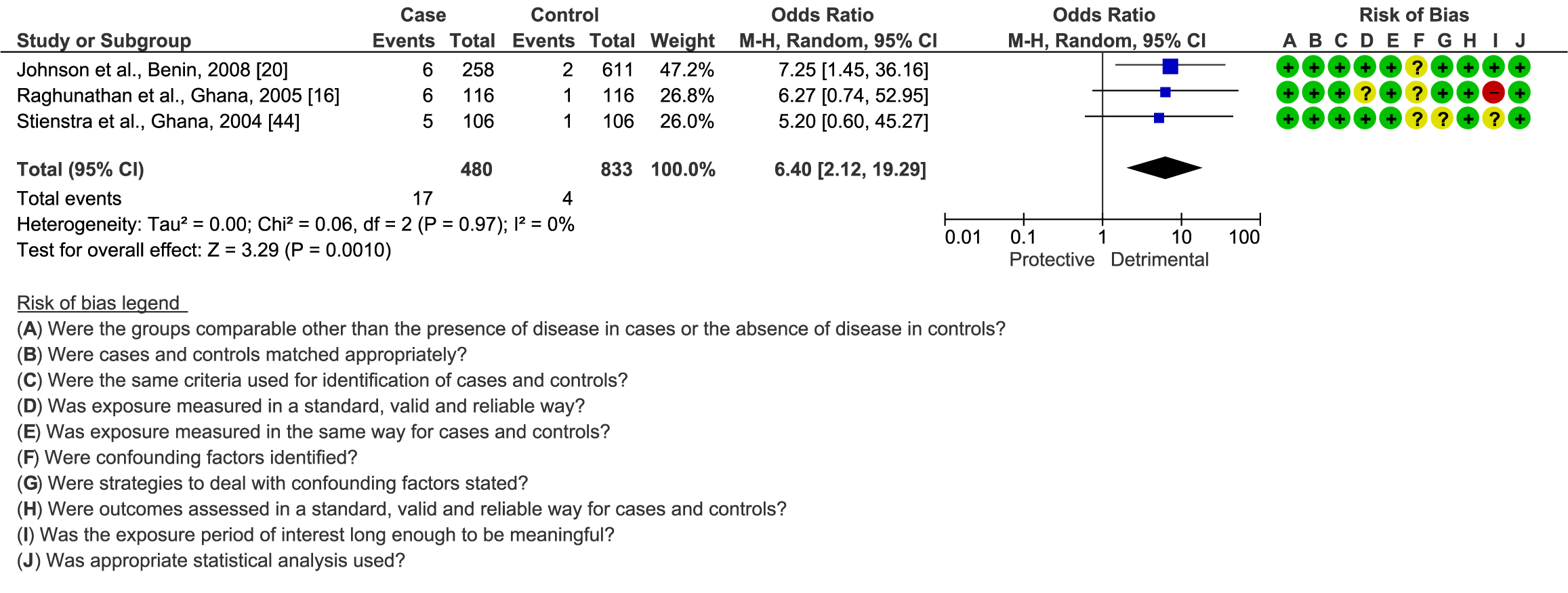

Supplement: S6 Fig — Bias: + low risk;—high risk; ? unclear risk. (TIF) [file pntd.0008161.s018.tif]

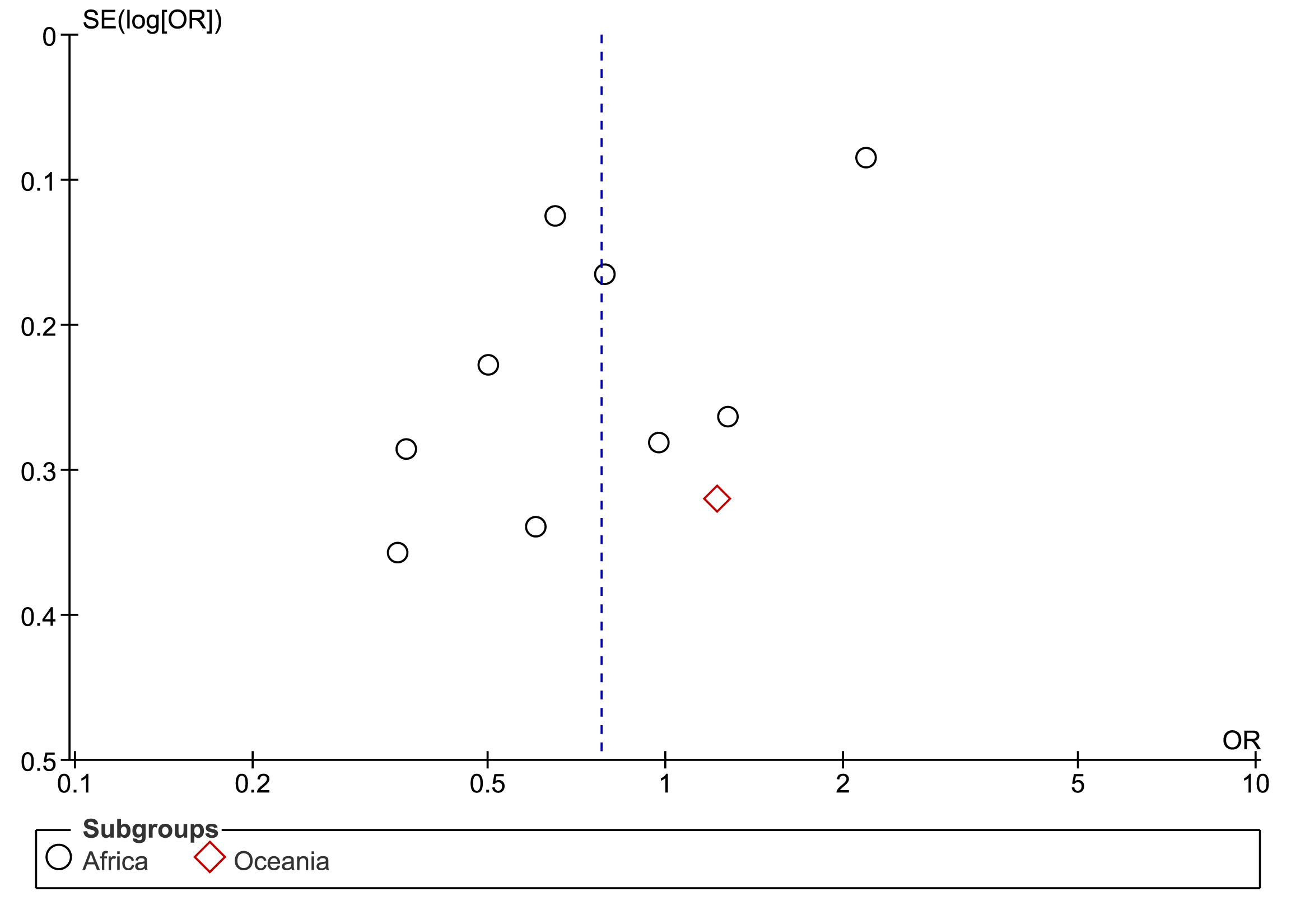

Supplement: S7 Fig — Each symbol is representative of a reference. SE–standard error. (TIF) [file pntd.0008161.s019.tif]
